# Cholesterol oxidase treatment impairs CXCR4-mediated T cell migration

**DOI:** 10.1186/s12964-025-02392-9

**Published:** 2025-10-17

**Authors:** Sofía R. Gardeta, Eva M. García-Cuesta, Blanca Soler Palacios, Rosa Ayala Bueno, Adriana Quijada-Freire, Noelia Santander Acerete, José Miguel Rodríguez-Frade, Mario Mellado

**Affiliations:** Chemokine Signaling group, Department of Immunology and Oncology, Centro Nacional de Biotecnología/CSIC, Campus de Cantoblanco, Madrid, E-28049 Spain

**Keywords:** Chemokines, Receptor conformations, CXCR4, Cholesterol, Lateral mobility, Cell migration

## Abstract

**Background:**

Cholesterol, a key component of mammalian cell membranes, modulates the properties of the lipid bilayer and influences the conformational states of membrane receptors, including G protein-coupled receptors (GPCRs). These effects are mediated through direct interactions with specific residues within the transmembrane regions and modulation of the surrounding lipid bilayer. Chemokine receptors, a GPCR sub-family, adopt distinct conformations associated with specific cell functions. For example, CXCL12 triggers receptor clustering, essential for directional cell migration. However, the precise mechanisms by which cholesterol controls the spatial organization of these receptors remain unclear. This study investigated the role of cholesterol in modulating the chemokine receptor CXCR4.

**Methods:**

We used lipidomic analysis to measure cellular cholesterol levels, and raster image correlation spectroscopy to assess the impact of cholesterol depletion on membrane fluidity. CXCR4 nanoclustering and dynamics were examined using single-particle tracking in TIRF mode. CXCR4 dimer formation was evaluated by FRET and FLIM analyses, and directed cell migration was measured using microfluidic chemotaxis chambers. Receptor expression and ligand binding were determined by flow cytometry with specific antibodies and CXCL12-ATTO700. Additional assays included calcium flux, and western blotting for signaling molecules. Statistical analysis used unpaired t-tests, one-way ANOVA, and two-tailed Mann-Whitney tests.

**Results:**

Our findings demonstrate that moderate cholesterol depletion using cholesterol oxidase increases membrane fluidity, impairs T cell migration towards CXCL12 gradients, and enhances CXCL12-mediated β1-integrin activation. This treatment also induced alterations in CXCR4 conformation and spatial distribution, without significantly affecting ligand binding or other chemokine-mediated signaling pathways. Immunocytochemical analysis indicated that cholesterol oxidase primarily affected the largest CXCR4 clusters, with no significant impact on lipid-enriched microdomains.

**Conclusions:**

This study identifies cholesterol as a crucial regulator of CXCR4 lateral mobility and spatial organization, enabling cells to effectively sense chemoattractant gradients.

**Supplementary Information:**

The online version contains supplementary material available at 10.1186/s12964-025-02392-9.

## Background

Chemotaxis is crucial in various biological processes, including cellular morphogenesis, innate immunity, inflammation, and cancer cell metastasis [1–3]. Although primarily associated with chemoattractant proteins, chemotaxis is also modulated by other molecules such as cytokines and lipid mediators. For example, eicosanoids such as leukotriene B4 and prostaglandin D2 are key initiators of chemoattractant signaling cascades [4].

Membrane lipids also contribute significantly to chemotactic signaling, serving both as essential components of signal transduction pathways and as docking sites for signaling proteins within the cytoplasm. They also act as substrates for lipid kinases and phosphatases, and can be cleaved to produce bioactive molecules that function as ligands or intermediates in downstream signaling. For instance, phospholipase C cleaves the polar head group from phospholipids to generate diacylglycerol, a critical second messenger involved in activating T cells and guiding thymocyte differentiation [5, 6]. Furthermore, phosphoinositides such as PI(4,5)P_2_ and PtdIns(3,4,5)P_3_ are indispensable in signaling pathways that regulate actin polymerization, pseudopodia formation, and directional cell migration [7, 8].

The cell membrane is composed of a lipid bilayer, with hydrophilic head groups facing the cytoplasmic and extracellular environments, and hydrophobic tails oriented inward. This asymmetric arrangement of phospholipids not only forms a passive barrier and scaffold for membrane proteins, but also actively shapes the biophysical properties of the membrane and supports essential cellular functions [9]. Additionally, other lipids such as sphingomyelin, ceramides, and cholesterol are integral to the fluid mosaic structure of the membrane, regulating its fluidity and facilitating dynamic signaling processes. Notably, a prolonged breakdown of sphingomyelins can significantly alter membrane fluidity and impair the ability of cells to sense chemoattractant gradients [10].

Cholesterol, a major component of mammalian cell membranes, plays a critical role in regulating the activity of integral membrane proteins [11–13]. Perturbations in cholesterol distribution and metabolism, whether of genetic or environmental origin, are implicated in a range of pathologies, including Alzheimer’s disease [14] cardiovascular disease [15], and diabetes [16]. Furthermore, cholesterol is increasingly recognized as an allosteric modulator of G protein-coupled receptors (GPCRs) [17], influencing both their conformation and function [18, 19].

Cholesterol impacts GPCR function on several levels. It affects both receptor oligomerization [20, 21] and the assembly of signaling complexes [22]. Structural studies employing X-ray crystallography and cryoelectron microscopy have demonstrated direct interactions between cholesterol (or cholesterol analogs) and specific residues within the transmembrane (TM) domains of GPCRs [23], modulating their activity. Furthermore, cholesterol can indirectly affect GPCR function by altering the biophysical properties of the surrounding lipid bilayer [24].

Chemokines direct leukocyte trafficking to sites of action in both homeostatic and inflammatory contexts. They act by binding to specific GPCRs present on the surfaces of migrating cells. Compelling evidence suggests that chemokine receptors exist not only as monomers, but also as homo- and heterodimers, and even higher-order oligomers. These distinct oligomeric states contribute to the diverse functional repertoire of this important family of inflammatory mediators [25, 26]. Like all integral membrane proteins, chemokine receptors reside within a compartmentalized lipid bilayer, which significantly influences their dynamics [27]. Indeed, the association between CXCR4 and lipid-enriched membrane microdomains has been shown to be functionally important [28–30].

Cholesterol is known to influence chemokine receptor function [31], and in the case of CXCR4, a cholesterol molecule has been observed nestled between its two protomers, engaging in hydrophobic interactions between TM region residues [32].

Here, using CXCR4 and its ligand, CXCL12, as a model system, we employed single-particle tracking (SPT) with total internal reflection fluorescence (TIRF) microscopy to investigate the impact of moderate cholesterol depletion on CXCR4 nanoclustering and dynamics in T cells. We found that treating Jurkat cells with cholesterol oxidase (ChOx) resulted in a modest reduction in cellular cholesterol levels, with minimal changes in sphingomyelin/ceramide content. This manipulation increased membrane fluidity and abolished CXCL12-mediated directional cell migration, while enhancing β1-integrin activation. Notably, CXCL12 binding and other CXCL12-mediated functions remained unaffected. Fluorescence resonance energy transfer (FRET) and fluorescence lifetime imaging microscopy (FLIM) experiments revealed that ChOx treatment induced conformational changes in CXCR4 that were also evident after ligand binding. SPT/TIRF analysis showed that while CXCL12-induced CXCR4 clustering was maintained following ChOx treatment, the stabilization of larger clusters (containing ≥ 10 receptors/particle) was impaired. Finally, immunocytochemical staining for cholera toxin B (CTxB), a marker for lipid-enriched microdomains [33], showed no significant alterations after ChOx treatment. This observation is consistent with the preservation of CXCL12-mediated clustering, despite the evident disruption of the largest CXCR4 clusters.

## Materials and methods

### Cells and reagents

HEK-293T cells were obtained from the ATCC (CRL-11268) and human Jurkat leukemia CD4^+^ cells were kindly donated by Dr. J. Alcamí (Centro Nacional de Microbiología, Instituto de Salud Carlos III, Madrid, Spain). When needed, Jurkat cells lacking endogenous CXCR4 (JKX4^−/−^) [34] were transiently transfected with CXCR4-AcGFP (20 µg; JKX4^−/−^-X4) using a Gene Pulser Xcell (BioRad, Hercules, CA) electroporator (20 × 10^6^ cells/400 µL RPMI 1640 with 10% fetal calf serum) and analyzed 24 h later.

The following antibodies were used: anti-human CXCR4 (clones 44717 and 12G5) and anti-human CXCR4-PE (clone 12G5), all from R&D Systems (Minneapolis, MN); goat anti-mouse IgG (H + L)-PE from Southern Biotech (Birmingham, AL); anti-phospho-ERK1,2 (#9191), anti-ERK (#9102, #13038), anti-pAkt T308 (#13038), anti-pAkt S473 (#9271) and anti-Akt (#9272) from Cell Signaling Technology (Danvers, MA); and phalloidin-TRITC (#P195) from Sigma-Merck (Darmstadt, Germany). Human CXCL12 was purchased from PeproTech (Rocky Hill, NJ). Human CXCR4 was cloned into pECFP-N1, pEYFP-N1, and pAcGFPm-N1 vectors from Clontech Laboratories (Mountain View, CA), as described [25]. Bacterial sphingomyelinase (bSMase obtained from *Staphylococcus aureus*, S9396), ChOx (C5421), and methyl-β-cyclodextrin (MCD; 779873) were obtained from Sigma-Aldrich (Madrid, Spain). CellTrace CFSE and the Di-4-ANEPPDHQ probe were from Invitrogen (Carlsbad, CA). CXCL12-ATTO700 was kindly donated by Dr. Marcus Thelen (Institute for Research in Biomedicine, Bellinzona, Switzerland). The HUTS-4 antibody [35] was kindly donated by Dr. Carlos Cabañas (Centro de Biología Molecular Severo Ochoa, Madrid, Spain).

### Lipidomics analysis using UHPLC-MS

Lipidomics analysis was performed on 12 Jurkat cell samples, divided into four treatment groups: bSMase (0.5 U/mL, 3 h, 37 °C), ChOx (25 U/mL, 2 h, 37 °C), MCD (5 mM, 30 min, 37 °C) or control (untreated).

Lipids were extracted and fractionated based on their physicochemical properties using a series of organic solvents. A chloroform/methanol extraction was used to isolate apolar lipids, which were then analyzed by UHPLC-MS to profile glycerolipids, cholesterol esters, sphingolipids, and glycerophospholipids. LC-MS-grade solvents were purchased from Sigma-Aldrich (St. Louis, MO) and Fisher Scientific (Pittsburgh, PA). Reference metabolite standards were obtained from Sigma-Aldrich, Avanti Polar Lipids (Alabaster, AL), and Larodan Fine Chemicals (Malmö, Sweden).

Cells (7.5 × 10^6^) were resuspended in cold water and briefly mixed. Proteins (380–454 µg/mL) were then precipitated by adding a cold chloroform/methanol solution to the lysed cells. Samples were incubated for 30 min at −20 °C and then vortexed. Then, different fractions of these samples were obtained and the following procedures were applied to extract the target analyte chemical class (glycerolipids, cholesteryl esters, sphingolipids, and glycerophospholipids). Cold water was added to a 500 µL fraction of the sample, which was then incubated for an additional 30 min at −20 °C. Subsequently, samples were vortex-mixed and centrifuged (18,000 × g, 15 min, 4 °C) to facilitate the separation of the organic and aqueous phases. The organic layer was collected, dried under vacuum, and resuspended in acetonitrile/isopropanol (1:1). Samples were then transferred to vials for UHPLC-MS analysis. Chromatography was performed using an ACQUITY UPLC system (Waters Corp., Milford, MA), coupled to a Xevo G2 Q-Tof mass spectrometer (Waters Corp.). Sample injections were randomized, with calibration and validation extracts uniformly interspersed throughout the entire batch run. Raw data are available at 10.21228/M80R8H, Metabolomics Workbench, project PR002318 [36].

### Western blotting

Cells (3 × 10^6^) were activated with CXCL12 (50 nM) at the indicated time points and then lysed in RIPA detergent buffer supplemented with 1 mM PMSF, 10 µg/mL aprotinin, 10 µg/mL leupeptin, and 10 µM sodium orthovanadate for 30 min at 4 °C. Extracts were analyzed by western blotting using specific antibodies.

### Flow cytometry studies

Cells were incubated with specific antibodies (30 min, 4 °C) and mean fluorescence intensity was determined on a FC500 flow cytometer (Beckman Coulter Inc., Brea, CA). Receptor internalization was determined by flow cytometry after activation with CXCL12 (50 nM) at the indicated time points. Results are expressed as a percentage of the mean fluorescence intensity of treated cells relative to that of unstimulated cells.

To assess β1-integrin activation, 3 × 10^5^ Jurkat cells, treated or not with ChOx (25 U/mL, 2 h, 37 °C), were plated in 50 µl of RPMI medium containing 1% BSA and 20 mM HEPES (Biowest, Nuaillè, France) in a 96-well V-bottom plate, and were stimulated or not with 50 µl of CXCL12 (50 nM) for 15 min at 37 °C in the presence 1.5 µg/100 µL of HUTS-4 Ab, an antibody that recognizes the active form of β1-integrins [35]. As a control, cells were also stained with an anti-CD29 antibody (1.5 µg/100 µL) to determine the presence of total β1-integrins. The cells were then washed at 4 °C once with 100 µl RPMI and once with 200 µl PBS. Goat anti-mouse IgG (H + L)-PE secondary antibody was then added in PBS (30 min at 4 °C in the dark). Subsequently, following two washes with PBS, cells were fixed with 2% paraformaldehyde (10 min, 4 °C) and washed twice more with PBS. Finally, samples were analyzed using a CytoFLEX cytometer (Beckman Coulter).

### CXCL12-ATTO700 binding

Untreated or treated Jurkat cells (2 × 10^6^ cells/mL) were incubated with CXCL12-ATTO700 (0.05 µM, 30 min, 37 °C) in culture medium without fetal calf serum. After washing, staining was analyzed by flow cytometry using CytoFLEX cytometer (Beckman Coulter).

#### Annexin-V/PI staining

Untreated and treated Jurkat cells (1 × 10^6^) were collected and washed twice in PBS prior to staining with ApoScreen Annexin-V FITC (Southern Biotech)/Propidium Iodide (PI) (Beckman Coulter). Briefly, cells were resuspended in 100 µl ApoScreen Annexin V Binding Buffer and 10 µl of Annexin-V-FITC. Cell suspensions were vortexed and incubated (15 min in darkness, 4 °C), and 380 µl ApoScreen Annexin V Binding Buffer with 10 µl of PI was added to each sample without a washing step. Samples were immediately analyzed using flow cytometry (Gallios Flow Cytometer, Beckman Coulter). As a control for cell death, Jurkat cells were treated with H_2_O_2_ (10%, 5 min, 37 °C). Results are expressed as the percentage of Jurkat cells.

### Transwell migration assay

Jurkat cells were transiently transfected with a constitutively active AKT (HA PKB^T308D/S473D^) (Addgene, Watertown, MA) or the empty vector (pcDNA3). 24 h later, transfected cells (3 × 10^5^) in 0.1 mL of RPMI containing 10 mM HEPES and 0.1% BSA were placed in the upper chamber of fibronectin (20 µg/mL, 60 min, 37 °C; Sigma)-coated transwells (5 μm pore; Costar, Corning, NY). CXCL12 (2.5 nM, 12.5 nM) in 0.6 mL of the same medium was added to the lower chamber. Plates were incubated for 120 min (37 °C, 5% CO_2_) and cells that migrated to the lower chamber were counted by flow cytometry (FC500, Beckman Coulter Inc., Brea, CA), corrected for variations in input cell concentrations, and expressed as the mean (standard deviation, SD) percentage of cell migration.

### Migration on planar lipid bilayers

Planar lipid bilayers were prepared as described previously ([37, 38]). Briefly, unlabeled and biotinylated DOPC liposomes were mixed at an specific ratio to obtain the desired molecular density for VCAM1 (300 molecules/µm^2^). Membranes were assembled onto sulphochromic solution-treated glass coverslips (Ibidi, Martinsried, Germany) affixed with adhesive 6-lane chambers (Ibidi), blocked with PBS/2% FCS (1 h, room temperature). Monobiotinylated recombinant human VCAM1-Fc (Biolegend, San Diego, CA) was tethered to membranes by incubation with AlexaFluor647-streptavidin (Invitrogen). Monobiotinylation was achieved labeling the antibody with 1 µg/ml NHS-LC-LC-Biotin (30 min, RT, in PBS; Thermo Fisher Scientific, Waltham, MA), followed by dialysis and checked by FACS. Finally, membranes were coated or not with CXCL12 (200 nM, 30 min, room temperature).

Cells (2 × 10^6^ cells/mL) in PBS containing 0.5% FCS, 0.5 g/l D-glucose, 2 mM MgCl_2_, and 0.5 mM CaCl_2_ were then infused into the prewarmed chamber (37 °C). Confocal fluorescence, differential interference contrast (DIC) and interference reflection microscopy (IRM) images were acquired on a Time-lapse images of cells were acquired on a Leica TCS SP5 inverted confocal microscope (Leica Microsystems GmbH, Wetzlar, Germany) fitted with a 100× oil-immersion objective (HCX PL APO 100×/1.47 NA). ImageJ 1.49v were used for qualitative and quantitative analysis of cell dynamics parameters, fluorescence and IRM signals. We calculated the frequency of migration (cells showing and IRM^+^ contact and moving over time).

#### Directional cell migration

Ibidi µ − Slide Chemotaxis chambers (ref: 80326; Ibidi) were first coated with fibronectin (20 µg/mL, 60 min, 37 °C; Sigma). Jurkat cells, either untreated or treated with ChOx (25 U/mL, 60 min, 37 °C), were diluted to 10 × 10^6^ cell/mL in RPMI medium containing 1% BSA and 20 mM HEPES in the presence or not of ChOx (25 U/mL). These cells were seeded into the channel of the chemotaxis slide and cultured (60 min, 37 °C). The reservoirs were then filled with chemotaxis medium in the presence or not of 25 U/mL ChOx, and 50 nM CXCL12 was added to the right reservoir. Phase-contrast images were recorded over 20 h with a time lapse of 2 min using a Leica Microfluor inverted microscope with a 10× objective (Leica Microsystems) and equipped with an incubation system set to 5% CO_2_ and 37 °C. Single-cell tracking was evaluated by selecting the center of mass in each frame using the manual tracking plug-in tool in ImageJ (NIH, Bethesda, MD). Spider plots, representing the trajectories of the tracked cells, forward migration index (FMI), and straightness values were obtained using the chemotaxis and migration plug-in tool (https://ibidi.com/chemotaxis-analysis/171-chemotaxis-and-migration-tool.html, Ibidi).

#### Static cell adhesion

Jurkat cells transiently transfected with a constitutively active AKT (HA PKB^T308D/S473D^) (Addgene, Watertown, MA) or the empty vector (pcDNA3) (3 × 10^5^) were labeled with CellTrace CFSE (1 mM, 10 min, 37 °C) and seeded onto a 96-well plate precoated with fibronectin (20 µg/mL) alone or with CXCL12 (125 nM) in RPMI medium containing 1% BSA and 20 mM HEPES. Cells were incubated (5 min, 37 °C) and washed twice. Finally, 100 µL/well of 0.5% SDS in PBS was added and the emission light was quantified using a Wallac Envision 2104 Multilabel Reader (Perkin Elmer, Foster City, CA).

#### Raster image correlation spectroscopy analysis

Cells were seeded (30 min, 37 °C) on Ibidi µ-well-chambers coated with fibronectin (20 µg/mL, 30 min, 37 °C). Adhered cells were untreated or treated with ChOx (25 U/mL, 2 h, 37 °C) prior to imaging. The Di-4-ANEPPDHQ probe was added (5 µM) to wells just before imaging. Cells were imaged in phenol-free medium supplemented with 10 mM HEPES and 0.1% BSA, at 37 °C and 5% CO_2_.

Time-lapse images of cells were acquired on a Leica TCS SP5 inverted confocal microscope (Leica Microsystems) fitted with an HCX PL APO 63×/1.2 NA water immersion objective. Di-4-ANEPPDHQ was excited using an argon white light laser at 488 nm. Emission signals were collected with two photomultiplier tubes (500–580, 620–750) and the pinhole was set to one Airy unit. Optimized acquisition was performed to retrieve membrane diffusion values, as described [39, 40]. Images of 256 × 256 pixels at 8-bit depth were collected using 80.4 nm pixel size and 4 µs dwell time, for 200 consecutive frames.

Raster image correlation spectroscopy (RICS) analysis was performed with “SimFCS 4” software (Global Software, G-SOFT Inc., Champaign, IL), as described [41]. RICS analysis was performed in regions of interest of 64 × 64 pixels at 4 random membrane areas per cell using a moving average (background subtraction) of 10 to discard possible artefacts due to cellular motion and slow-moving particles passing through. The autocorrelation 2D map was then fitted to obtain a surface map that was represented as a 3D projection with the residuals on top. As a general rule, we focused in those regions with intensity fluctuation events in which the intensity changes were following short increasing or decreasing steps, avoiding abrupt intensity decays or increases.

### ***Total internal reflection fluorescence analysis***

JKX4^−/−^ cells were transiently transfected with the CXCR4 receptor fused to the AcGFP monomeric protein (JKX4^−/−^-X4). Twenty-four hours after transfection, cells expressing low receptor-AcGFP levels were selected by cell sorting on a FACSAria Fusion platform (BD Biosciences, Franklin Lakes, NJ) for detection and tracking analysis. CXCR4 expression levels were then evaluated using the DakoCytomation Qifikit (Glostrup, Germany) and flow cytometry, as described [42]. Transfected cells expressing ~ 8,500–22,000 receptors/cell, which equates to a density of < 4.5 particles/µm^2^, were selected for detection and tracking analysis.

Experiments were performed with cells untreated or treated with ChoX (25 U/mL, 2 h, 37 °C) using a TIRF microscope (Leica AM TIRF inverted) equipped with a Hamamatsu Flash 4 digital sCMOS camera (Hamamatsu Photonics Europe GmbH, Herrsching, Germany), a 100× oil-immersion objective (HCX PL APO 100×/1.47 NA), and a 488-nm diode laser. The microscope was equipped with an incubator and temperature control units; experiments were performed at 37 °C with 5% CO_2_. Image sequences of individual particles (500 frames) were then acquired at 3.5% laser power with a frame rate of 10 Hz (90 ms/frame). Penetration depth of the evanescent field was 90 nm.

Particles were detected and tracked using described algorithms (U-Track2; [43] implemented in MATLAB, as described [44]. Mean spot intensity (MSI), number of mobile and immobile particles and diffusion coefficients (D_1-4_) were calculated from the analysis of thousands of single trajectories over multiple cells (statistics provided in the respective figure captions), using described routines [25, 45]. Receptor number along individual trajectories was determined as described [25, 46]. We measured the average fluorescence intensity for the first 20 frames of each trajectory and used the intensity of the monomeric protein CD86-AcGFP as a reference in JKX4^-/-^ cells transiently transfected with CD86-AcGFP. Distribution of monomeric particles intensities was analyzed by Gaussian fitting, rendering a mean value of 69.33 ± 3.26 a.u. A similar intensity value of the monomer was obtained for CXCR4-AcGFP particles showing a unique photobleaching step. Therefore, this value was used as the monomer reference to estimate the number of CXCR4-AcGFP molecules per particle [10].

#### Fluorescence resonance transfer analysis by sensitized emission

HEK-293T cells transiently transfected at a fixed CXCR4-YFP: CXCR4-CFP ratio (15 µg : 9 µg, respectively) or 5HTR_2B_-YFP: CXCR4-CFP (15 µg : 9 µg, respectively) as a negative control, were untreated or treated with ChOx (25 U/mL, 2 h, 37 °C) and FRET efficiency was evaluated in the absence or presence of CXCL12 (100 nM, 30 min). Emission light was quantified using the Wallac Envision 2104 Multilabel Reader equipped with a high-energy xenon flash lamp (donor: receptor fused to C-CFP, 8-nm bandwidth excitation filter at 405 nm; acceptor: receptor fused to YFP, 10 nm bandwidth excitation filter at 510 nm).

#### Fluorescence lifetime imaging

FLIM was determined in live, transiently-transfected HEK-293T cells with a fixed CXCR4-YFP/CXCR4-CFP ratio (15 µg : 9 µg, respectively) or 5HTR_2B_-YFP: CXCR4-CFP (15 µg : 9 µg, respectively) as a negative control, cultured in coverslip chambers (Nunc, Naperville, IL). As reference for CFPτ, HEK-293T cells were transiently transfected with CXCR4-CFP (9 µg). Cells were first left untreated or treated with ChOx (25 U/mL, 2 h, 37 °C) and then stimulated or not with CXCL12 (100 nM, 30 min). FLIM measurements were performed on a Leica STELLARIS 8 STED 3X Falcon DLS confocal microscope with a high-speed lifetime module and an HC PL APO CS2 1.2 NA 63× water immersion objective. Fluorescence lifetime (CFPτ and YFPτ) was measured after excitation with a white laser line at 448 nm with a 448 nm notch filter, and emission was collected with a HyD X2 detector at 460–495 nm and a HyD X4 at 518–605 nm in intensity mode. Data were analyzed using Leica LasX FLIM/FCS software. Lifetime values were obtained for 5 independent experiments of at least 20 cells each.

#### Immunofluorescence analyses

Jurkat cells, treated or not with ChOx (25 U/mL, 2 h, 37 °C), were plated on fibronectin (20 µg/mL, Sigma)-coated glass slides and were then stimulated or not with 100 nM CXCL12 (5 min at 37 °C) and fixed with 4% paraformaldehyde (10 min, room temperature [RT]). Preparations were blocked with PBS containing 150 mM NaCl, 0.1% goat serum, and 1% BSA (60 min, RT) before staining with anti-human ICAM-3 plus AlexaFluor 488 goat anti-mouse IgG (30 min, RT, Thermo Fisher Scientific). Cells were permeabilized with 0.25% saponin (10 min, RT) and stained with phalloidin-TRITC (Sigma-Merck; 30 min, RT). Preparations were analyzed using a ZEISS confocal multispectral microscope (Jena, Germany).

#### Cholera toxin B staining

Jurkat cells, untreated or treated with ChOx (25 U/mL, 2 h, 37 °C) or bSMase (0.5 U/mL, 3 h, 37 °C) were plated on fibronectin (20 µg/mL, Sigma)-coated glass slides and were fixed with 4% paraformaldehyde (10 min, RT). Preparations were blocked with PBS containing 150 mM NaCl, 0.1% goat serum, and 1% BSA (60 min, RT) before staining with anti-human CXCR4 (clone 44717) plus AlexaFluor 546 goat anti-mouse IgG (30 min, RT, Thermo Fisher Scientific). Cells were then washed and stained with 12 µg/µl CTxB-FITC (Sigma-Merck; 15 min, RT). Samples were analyzed using a LEICA Stellaris 5 multispectral confocal microscope.

#### Statistical analysis

Data were analyzed using GraphPad PRISM (ns = not significant *p* > 0.05; *p ≤ 0.05; ** *p* ≤ 0.01; *** *p* ≤ 0.001; **** *p* ≤ 0.0001). Most experiments were analyzed using Student’s t-test. Data from experiments of internalization, actin polymerization and western blotting were analyzed using two-way ANOVA followed by Tukey’s multiple comparison test. MSI was analyzed using one-way ANOVA followed by Tukey’s multiple comparison test. A two-tailed Mann-Whitney non-parametric test was used to analyze the diffusion coefficient (D_1−4_) of single particles.

For lipidomic analysis, statistics were analyzed using Matlab (R2018a, MathWorks), by the Mann-Whitney U test (*p* ≤ 0.05) after normality testing with the Shapiro-Wilk test. The Benjamini-Hochberg correction was used to control the false discovery rate at α = 0.05.

## Results

### ChOx treatment affects the levels of cholesterol in Jurkat cells

Methyl β-cyclodextrin (MCD) has been widely used to investigate the role of cholesterol in GPCR biology, but its disruptive effects limit its utility. Cyclodextrins can extract cholesterol from both raft and non-raft membrane domains, redistribute cholesterol between plasma and intracellular membranes, and even remove other hydrophobic molecules, such as phospholipids [10]. Because MCD treatment is known to affect both ligand binding and various CXCR4 functions [30], we first examined its impact on CXCR4 expression in our cell system using flow cytometry with specific antibodies. Our results showed that MCD severely impaired cell viability and markedly affected the binding of two conformation-sensitive anti-CXCR4 monoclonal antibodies (mAbs) in live cells (Supplementary Fig. 1a, b). Consequently, we chose ChOx as a more gentle and specific method for modulating cholesterol levels in the cell membrane. ChOx specifically oxidizes cholesterol within lipid rafts to cholestenone, and this oxidation does not inhibit the activation of either Jurkat cells or CD4^+^ T lymphocytes [10].

**Fig. 1:**
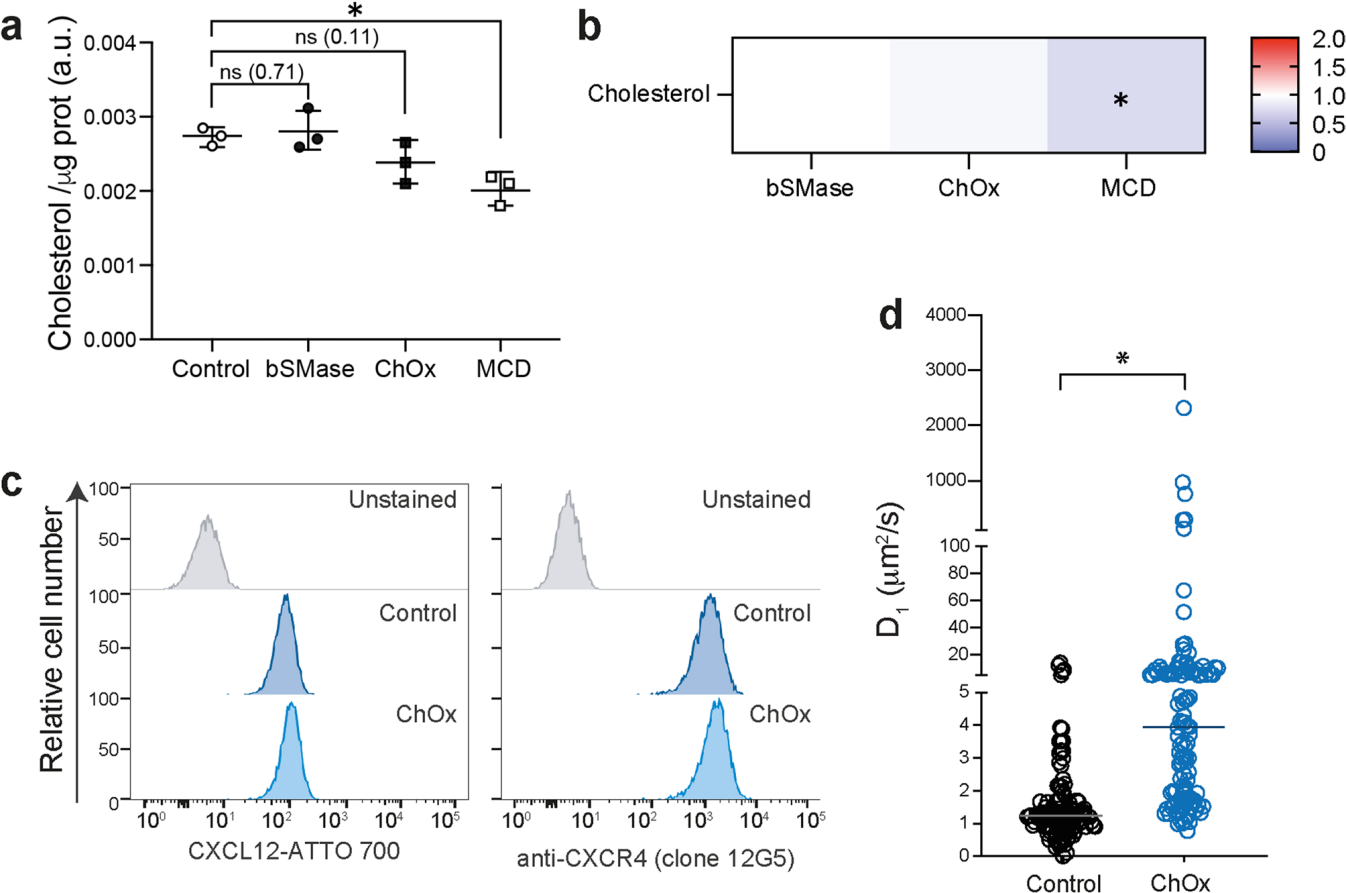
Effect of cholesterol oxidase treatment on cholesterol levels, CXCR4 expression, ligand binding, and membrane fluidity in Jurkat cells. **a** UHPLC-MS analysis of cholesterol levels in Jurkat cells untreated (control) or treated with sphingomyelinase (bSMase), cholesterol oxidase (ChOx), or methyl-β-cyclodextrin (MCD). Figure shows the relative amount of cholesterol per µg of protein (a.u.). **b** Heatmap representing binary comparisons of Jurkat cells treated with bSMase, ChOx and MCD compared with the control group. Heatmap color codes for log_2_ (fold-change). Student’s t-test *n* = 4; n.s.: not significant; ∗*p* ≤ 0.05. **c** CXCL12 binding to untreated and ChOx-treated Jurkat cells analyzed by flow cytometry using CXCL12-ATTO700. Data show a representative experiment of 5 performed (left panel). CXCR4 expression on the surface of untreated or ChOx-treated Jurkat cells analyzed by flow cytometry using an anti-CXCR4 monoclonal antibody (clone 12G5) (right panel). **d** RICS analysis was performed using the Di4-ANEPPDHQ probe at the plasma membrane of untreated and ChOx-treated Jurkat cells. Diffusion values obtained by RICS from untreated and ChOx-treated cells, mean is indicated (red) (*n* = 3, with at least 10 cells analyzed per experiment and condition; *p ≤ 0.05)

Lipidomic analysis using UHPLC-ESI-MS revealed that treatment with ChOx resulted in a modest decrease in cellular cholesterol levels, with no significant changes in most of the sphingomyelin and ceramide content (Fig. 1a, b and Supplementary Fig. 2). However, we observed a modest but significant decrease in some sphingomyelin d28 species that did not correlate with the expected increase in corresponding ceramides (Supplementary Fig. 2). By contrast, treatment with bSMase (positive control for sphingomyelin breakdown) led to a complete loss of sphingomyelins (Supplementary Fig. 2a, b) without affecting cholesterol levels. As anticipated, treatment with MCD (positive control for cholesterol downregulation) induced a substantial decrease in cholesterol (Fig. 1a, b), consistent with the observed structural changes in CXCR4 (Supplementary Fig. 1b).

ChOx treatment did not compromise cell viability, as confirmed by flow cytometry analysis of Annexin V and PI staining (Supplementary Fig. 3). Furthermore, ChOx treatment did not alter either CXCR4 membrane expression or its binding for CXCL12, as assessed by flow cytometry using an anti-CXCR4 mAb and CXCL12-ATTO700, respectively (Fig. 1c).

### ChOx treatment affects cell membrane dynamics and some CXCR4-mediated functions

Cholesterol is an important component of mammalian cell membranes, influencing their fluidity and permeability. While cholesterol generally exhibits a condensing effect on fluid lipid membranes, its specific impact is complex and depends on the ratio of saturated to unsaturated lipids. Specifically, cholesterol stiffens membranes composed of saturated lipids, but does not have this stiffening effect on membranes containing unsaturated lipids [10].

Membrane fluidity was assessed using RICS with the fluorescent lipid probe Di-4-ANEPPDHQ, which also reports on lipid lateral packing [39, 40]. Jurkat cells, untreated or treated with ChOx, were labeled with Di-4-ANEPPDHQ and analyzed by RICS after confocal microscopy. Results indicated that membrane diffusion was higher in cells treated with ChOx than in untreated controls (Fig. 1d), suggesting that ChOx treatment increases membrane fluidity.

**Fig. 2:**
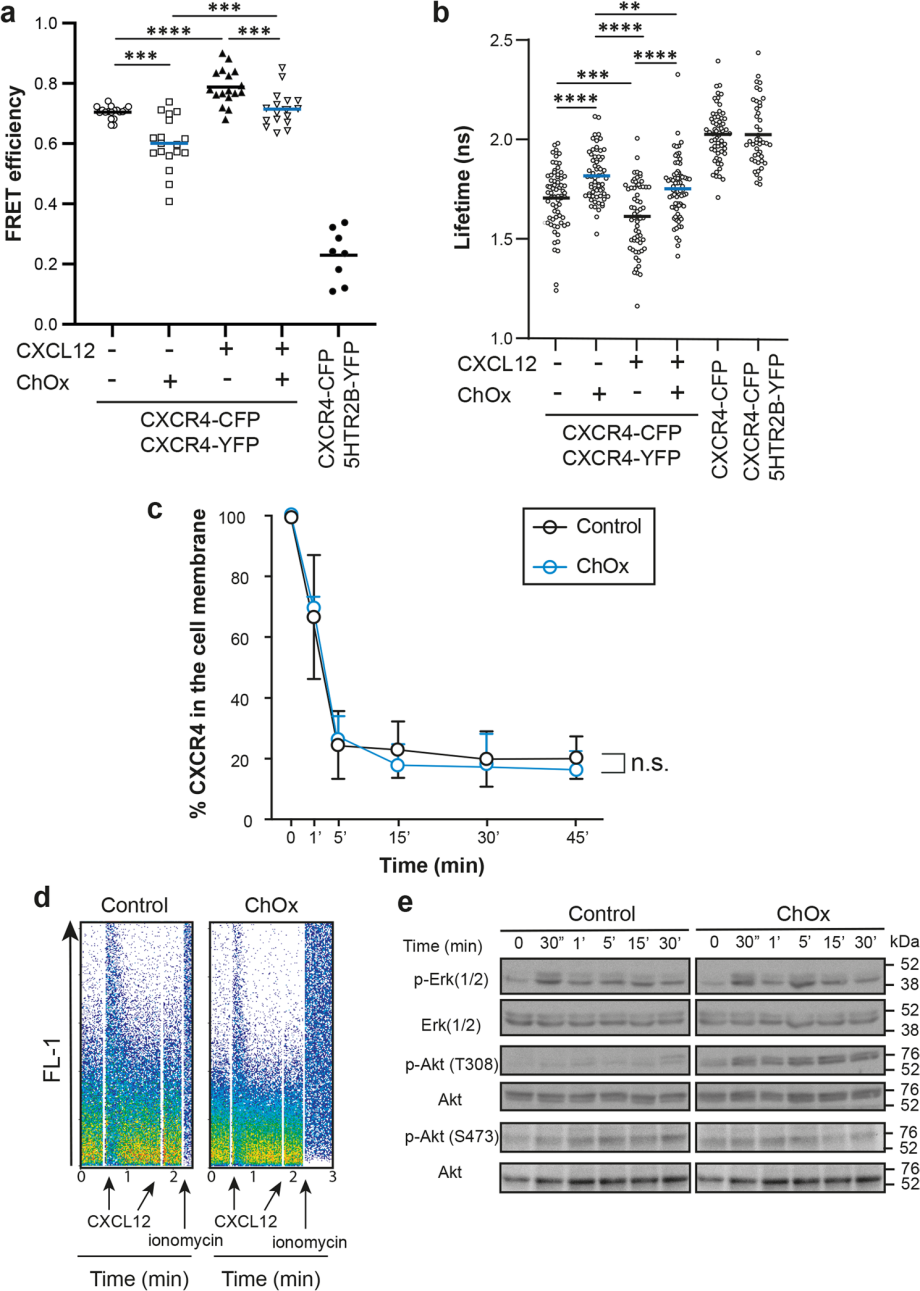
Effect of cholesterol oxidase treatment on CXCL12-mediated signaling. **a** CXCR4 conformation in the cell membrane is altered by cholesterol depletion. HEK-293T cells transiently transfected at 15:9 CXCR4-YFP: CXCR4-CFP ratio were untreated or treated with ChOx, stimulated or not with CXCL12 (50 nM), and FRET efficiency was determined. As a negative control, HEK-293T cells transiently transfected at 15:9 5HTR_2B_-YFP: CXCR4-CFP ratio were used; *n* = 3, mean ± SEM, ***p ≤ 0.001, ****p ≤ 0.0001. **b** CFP fluorescence lifetime (CFPτ) was determined in cells as in a) treated or not with ChOx and activated or not with CXCL12 (50 nM). As a reference, CFPτ in HEK-293T cells transiently transfected with CXCR4-CFP was evaluated. HEK-293T cells transiently transfected with 5HTR_2B_-YFP: CXCR4-CFP were used as a negative control. *n* = 3, mean ± SEM, **p ≤ 0.01, ***p ≤ 0.001, ****p ≤ 0.0001. **c** Cell surface expression of CXCR4 in untreated and ChOx-treated Jurkat cells after stimulation with CXCL12 (50 nM) at different time points, analyzed by flow cytometry using an anti-CXCR4 antibody (clone 44717). Results show mean ± SEM of the percentage of CXCR4 expression at the cell surface (*n* = 4, n.s., not significant). **d** CXCL12-mediated Ca^2+^ flux in untreated or ChOx-treated Jurkat cells. The ionophore ionomycin was used as control. A representative flow cytometry plot of each condition is shown (*n* = 3). **e** Western blot analysis of ERK 1/2 and Akt phosphorylation at residues Thr308 and Ser473 in untreated and ChOx-treated Jurkat cells stimulated with CXCL12 (50 nM) at the indicated time points (*n* = 3). Membranes were reblotted with anti-Akt and anti-ERK antibodies as loading controls. A representative experiment is shown

Cholesterol also influences membrane receptor conformation [32, 47]. We thus performed FRET experiments on untreated or ChOx-treated HEK-293T cells transiently co-transfected with CXCR4-CFP and CXCR4-YFP or with CXCR4-CFP and 5HTR_2B_-YFP as a negative control. Results showed that FRET efficiency was lower in ChOX-treated cells than in untreated cells (Fig. 2a), confirming that cholesterol depletion alters CXCR4 dimer conformation. We also detected a CXCL12-mediated increase in FRET efficiency under both conditions (Fig. 2a), confirming that the ligand can still bind CXCR4 and modulate its conformation despite the ChOx-induced changes. These findings were further supported by FLIM microscopy, which measures the donor fluorescence lifetime (τ), a constant parameter for a given fluorophore under specific experimental conditions. Data using HEK-293T cells transiently transfected with CXCR4-CFP alone showed a baseline CFP lifetime (CFPτ) of 2.03 ns. This value was lower in cells transfected with CXCR4-CFP/CXCR4-YFP, in both untreated and ChOx-treated cells (1.7 ns vs. 1.82 ns, respectively). As a control, no variations in CFPτ were observed in cells transfected with CXCR4-CFP/5HTR_2b_-YFP (2.02 ns). These results confirmed that ChOx-treated cells had an altered conformation of CXCR4 homodimers. We also evaluated the effect of ligand activation on this process, and the results indicated that CXCL12 bound CXCR4 in both experimental conditions and was able to modulate the conformation of the complexes (CFPτ 1.61 ns in untreated cells + CXCL12 vs. 1.75 ns in ChOx-treated cells + CXCL12) (Fig. 2b). While ChOx treatment had no effect on CXCL12 binding to CXCR4 (Fig. 1c, left panel), we investigated its impact on CXCR4-mediated functions. Flow cytometry analysis of Jurkat cells stained with an anti-CXCR4 mAb ruled out differences in CXCR4 internalization (Fig. 2c). Similarly, CXCL12-triggered Ca^2+^ flux (Fig. 2d) and CXCL12-mediated Akt (S473) and ERK1/2 phosphorylation (Fig. 2e, Supplementary Fig. 4) were comparable between untreated and ChOx-treated Jurkat cells. However, ChOx treatment induced strong and sustained AKT phosphorylation at Thr308 (Fig. 2e, Supplementary Fig. 4).

### Prolonged ChOx treatment impairs CXCR4-mediated directed T cell migration

**Fig. 3:**
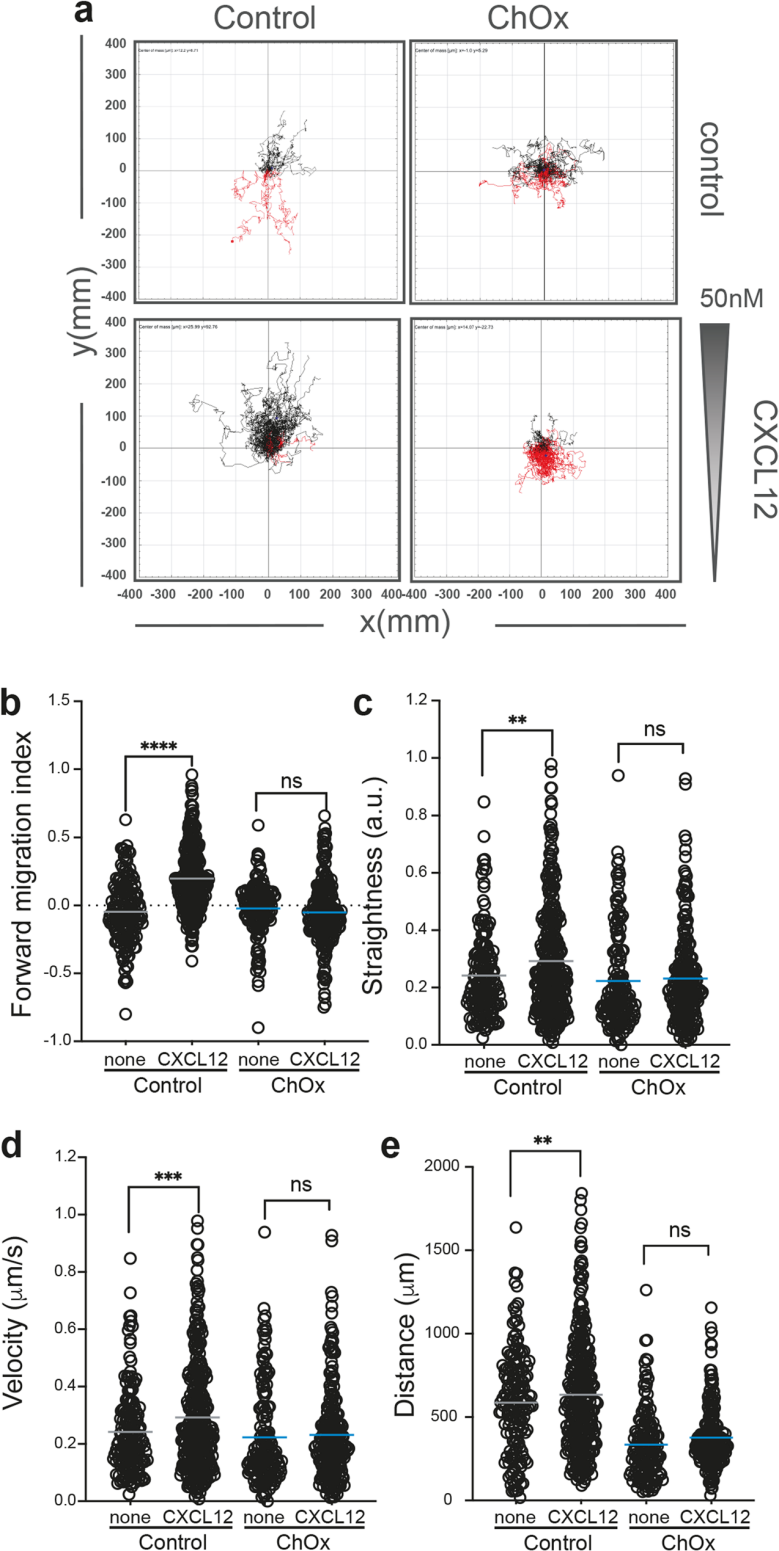
Effect of cholesterol oxidase treatment on CXCL12-mediated directed cell migration. Directional cell migration of untreated and ChOx-treated Jurkat cells on fibronectin-coated µ−slide-IBIDI chambers in response to CXCL12 (*n* = 5, in duplicate, with at least 50 different cell trajectories from each condition). **a** Representative spider plot showing trajectories of individual cells migrating toward the gradient (black lines) or moving in the opposite direction (red lines). The black and red dots represent the final position of each single tracked cell. **b-e** Quantification of forward migration index (**b**), track straightness (**c**), cell velocity (**d**), and displacement (**e**) of cells in (**a**). Figures show the data of individual cells, with the mean indicated (red) (n.s., not significant; **p ≤ 0.01; ***p ≤ 0.001; ****p ≤ 0.0001)

**Fig. 4:**
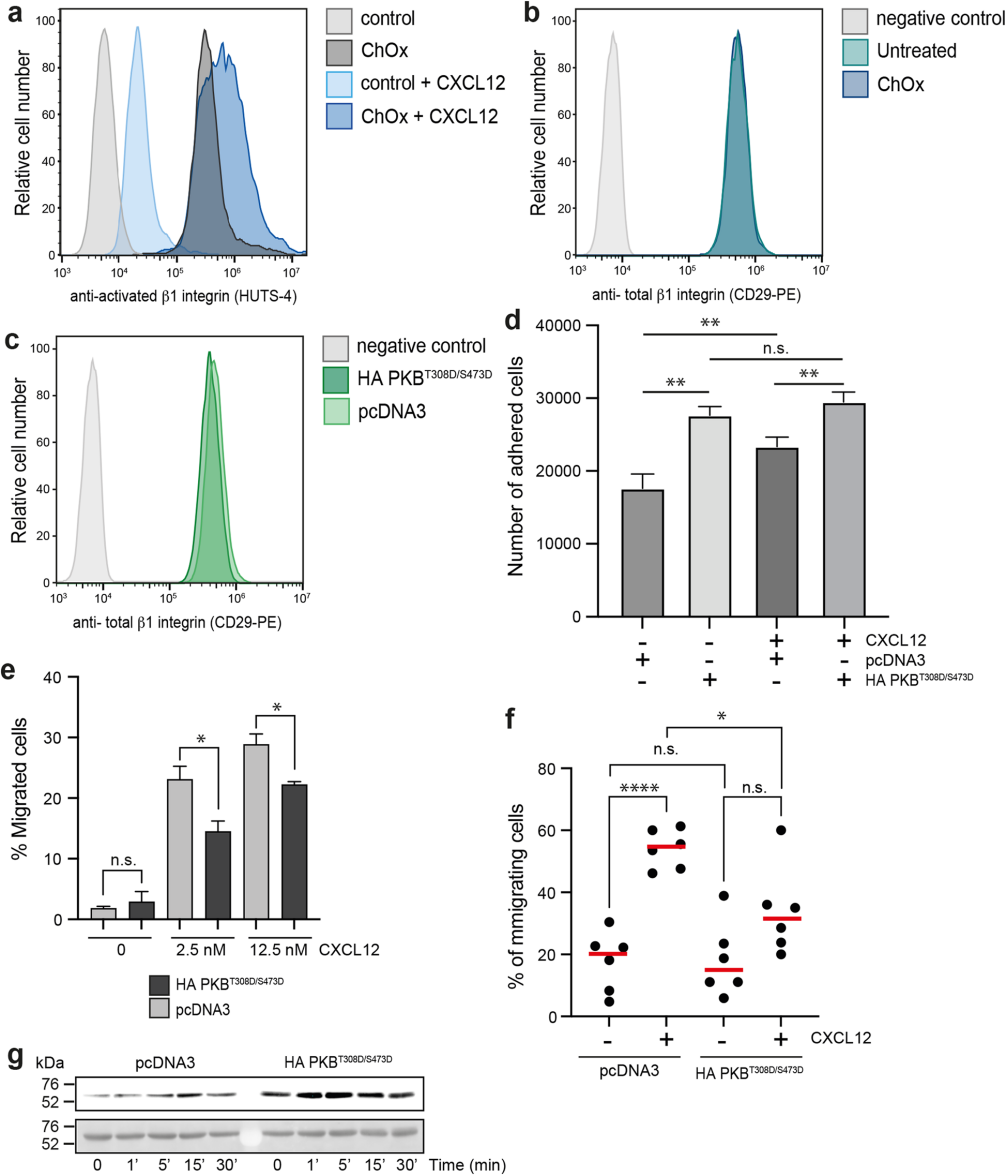
Effect of cholesterol oxidase treatment on CXCL12-mediated β1-integrin activation. (**a**) Expression and quantification of β1-integrin activation at the membrane of untreated and ChOx-treated Jurkat cells stimulated with CXCL12 (100 nM) analyzed by flow cytometry with the specific antibody HUTS-4. A representative experiment of 3 performed is shown. (**b**) Expression and quantification of total β1-integrin activation at the membrane of untreated and ChOx-treated Jurkat cells analyzed by flow cytometry with the specific antibody anti-CD29. A representative experiment of 3 performed is shown. (**c**) Expression of active β1-integrin at the membrane of untransfected and transiently-transfected Jurkat cells with the constitutively active AKT mutant (HA PKB^T308D/S473D^) or pcDNA3 (control) analyzed by flow cytometry with the specific antibody HUTS-4. A representative experiment of 3 performed is shown. (**d**) Jurkat cells as in c) were labeled with CFSE and seeded on wells coated with fibronectin (20 µg/mL) alone or with CXCL12 (125 nM). Figures show the fluorescence detected (a.u.), with the mean ± SD (*n* = 3; n.s., not significant; **p ≤ 0.01). (**e**) Migration of transfected cells as in c) determined using fibronectin-coated chambers in response to CXCL12 (2.5 nM and 12.5nM). Data are shown as the mean percentage (± SD) of input cells that migrate (*n* = 3; ns: not significant; *p ≤ 0.05). (**f**) Migration frequency of transfected cells as in c), was evaluated on lipid bilayers containing VCAM-1, either or in combination with CXCL12. Results are shown as mean ± SD (*n* = 6; ns: not significant; *p ≤ 0.05, ****p ≤ 0.0001). (**g**) Western blot analysis of Akt phosphorylation at residue Thr308 in transiently-transfected Jurkat cells with the constitutively active AKT mutant (HA PKB^T308D/S473D^) or pcDNA3 (control) stimulated with CXCL12 (50 nM) at the indicated time points (*n* = 3). Membranes were reblotted with anti-Akt antibodies as loading controls. A representative experiment is shown

We treated Jurkat cells with ChOx (25 U/mL, 2 h, 37 °C, 5% CO_2_) and then assessed their CXCL12-directed migration using fibronectin-coated µ − Slide chemotaxis chambers. Results showed that whereas untreated cells sensed CXCL12 gradients, ChOx-treated cells did not (Fig. 3a, Supplementary video 1, 2). Specifically, CXCL12 failed to induce increases in forward migration index, track straightness, velocity, or distance travelled in ChOx-treated cells (Fig. 3b-e). Further analysis of these data suggested that ChOx treatment increased cellular adhesion. ChOx-treated cells exhibited significantly reduced motility even in the absence of CXCL12 (Fig. 3d, e). This finding was corroborated by flow cytometry using the HUTS-4 mAb, which recognizes the active conformation of β1-integrins, with ChOx-treated cells showing a higher proportion of active β1-integrins than control cells (Fig. 4a). As a control, ChOx treatment did not alter the overall expression of total β1-integrins, as confirmed by flow cytometry using an anti-CD29 mAb (Fig. 4b).

These data collectively suggest that ChOx treatment inhibits CXCL12-mediated directed cell migration and enhances β1-integrin activation, while leaving other CXCR4-mediated signaling pathways unaffected. While the precise mechanism is not clear, we explored a potential link to the enhanced and sustained hyperphosphorylation of AKT at Thr308 induced by CXCL12 in cells treated with ChOx. Jurkat cells transiently transfected with HA PKB^T308D/S473D^, a constitutively active AKT mutant [48], exhibited similar levels of total β1-integrins (Fig. 4c) and showed enhanced CXCL12-induced adhesion to fibronectin when compared with control cells (Fig. 4d). Moreover, these transfected cells displayed reduced CXCL12-mediated migration in chemotaxis experiments on fibronectin-coated chambers (Fig. 4e) as well as in lipid bilayers coated with VCAM-1, relative to control cells transfected with the empty vector (Fig. 4f). Western blot analysis with an anti-pThr308-AKT antibody confirmed the strong and sustained AKT phosphorylation in cells transiently transfected with this constitutively active AKT mutant (Fig. 4g).

**Fig. 5:**
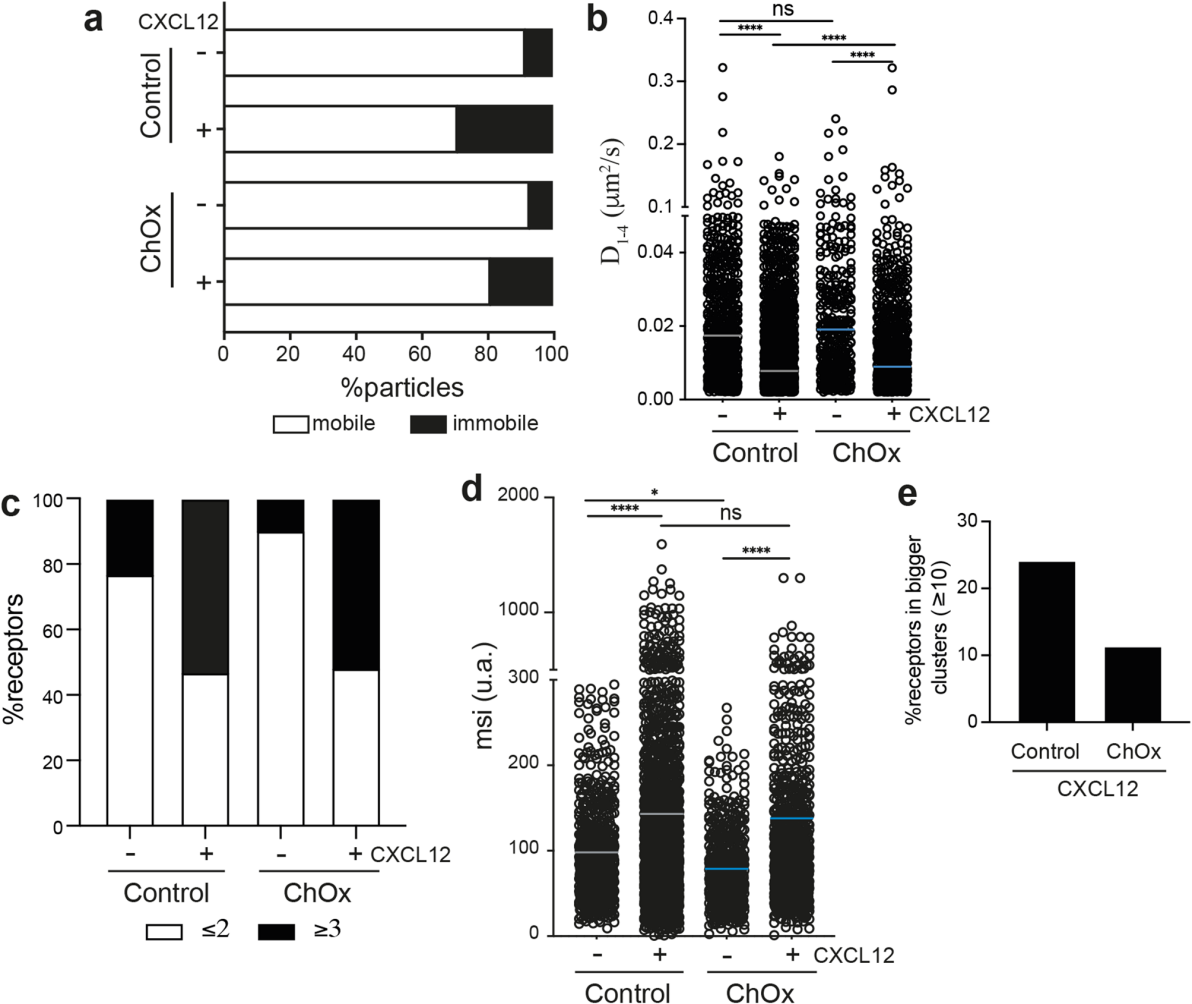
Cholesterol oxidase treatment does not alter CXCL12-mediated actin polymerization or cell polarization. **a** Flow cytometry analysis of polymerized actin by phalloidin-TRITC staining in untreated and ChOx-treated Jurkat cells stimulated with CXCL12 (50 nM) at different time points. A two-tailed paired Student’s t-test was performed for each time point (*n* = 3, mean ± SEM). **b** Representative confocal microscopy images of untreated and ChOx-treated Jurkat cells adhered to fibronectin and treated with CXCL12, as analyzed with an anti-ICAM-3-AlexaFluor 488 antibody (green) and phalloidin-TRITC, (red); figure also shows overlay of both channels with arrows indicating the leading edge (red) and the uropod (green). Scale bars, 10 μm. **c** Quantification of the percentage of polarized cells in the different experimental conditions evaluated in (B). Data correspond to the mean and variances (SEM) of 2 independent experiments with more than 150 cells analyzed in each condition (n.s., not significant; ****p ≤ 0.0001)

Actin cytoskeleton dynamics are crucial for cell movement, contributing not only to receptor compartmentalization through the formation and stabilization of protrusions or lamellipodia at the leading edge of motile cells, but also to the maintenance of directional migration [49]. Phalloidin staining and flow cytometry analysis demonstrated that ChOx treatment failed to affect CXCL12-mediated actin polymerization in Jurkat cells (Fig. 5a). Furthermore, confocal microscopy quantification of phalloidin and ICAM-3 staining in fixed cells showed that ChOx treatment did not impair CXCL12-mediated cell polarization (Fig. 5b, c).

**Fig. 6:**
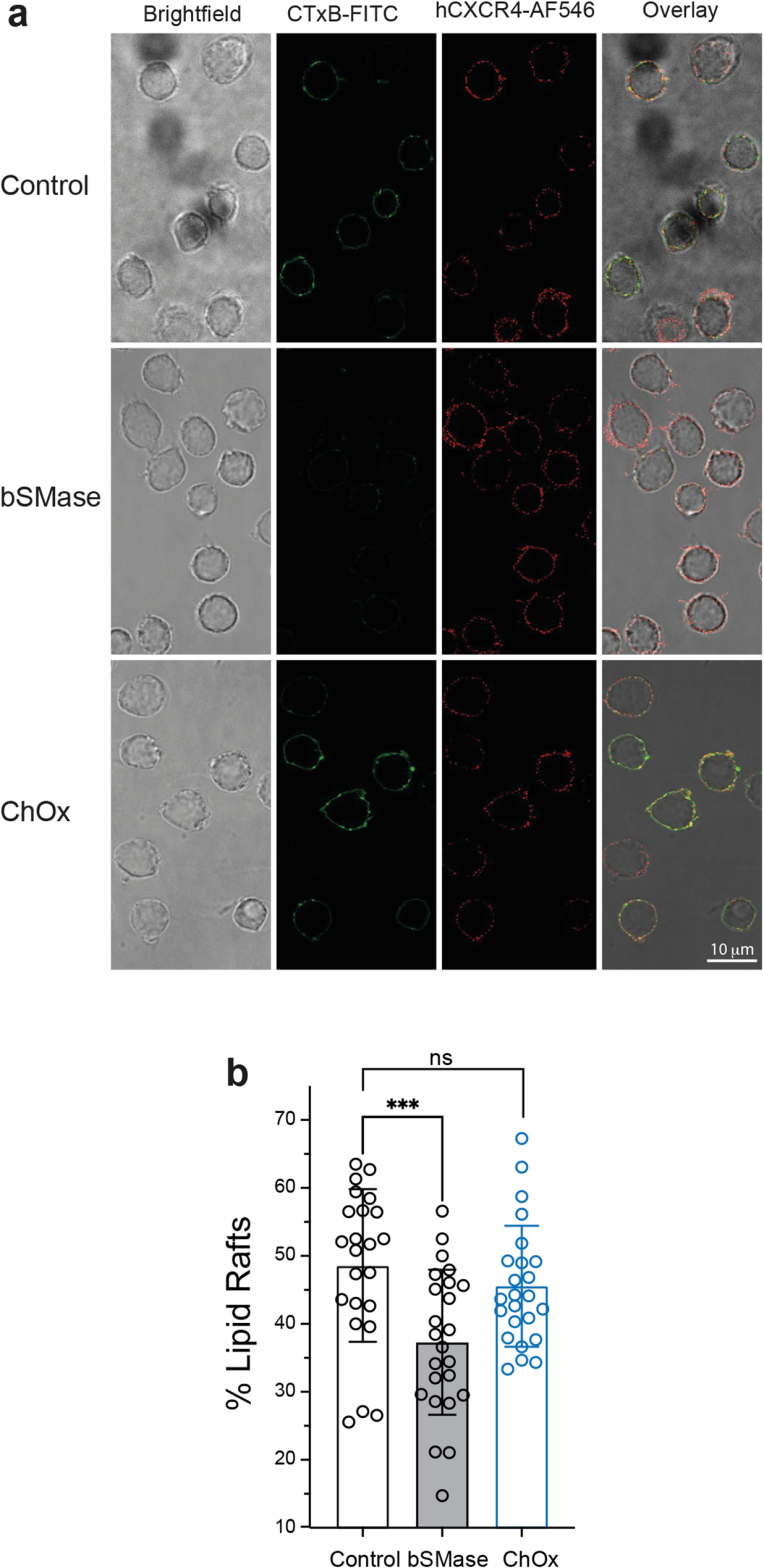
CXCR4 organization and dynamics at the cell membrane following cholesterol oxidase treatment. Single-particle tracking analysis (SPT) of JKX4^−/−^ cells transiently transfected with CXCR4-AcGFP untreated and treated with ChOx on fibronectin (FN) or FN + CXCL12-coated coverslips (untreated: 696 particles in 50 cells on FN; 1,736 in 53 cells on FN + CXCL12; ChOx-treated: 426 particles in 38 cells on FN, 901 in 50 cells on FN + CXCL12) (*n* = 2). **a** Percentage of mobile and immobile CXCR4-AcGFP particles at the membrane of cells treated as indicated. **b** Diffusion coefficients (D_1−4_) of the mobile cells treated as indicated, with median (black line) indicated. (n.s., not significant; ****p ≤ 0.0001). One-way ANOVA was performed followed by Tukey’s multiple comparison test for mean spot intensity (MSI), and a two-tailed Mann-Whitney non-parametric test for D_1−4_. **c** Frequency of CXCR4-AcGFP receptors present in monomeric and dimeric (≤ 2) or in oligomeric particles (≥ 3) expressed as a percentage and calculated by the MSI of each particle relative to the MSI value of the monomeric CD86-AcGFP protein. **d** Distribution of MSI (arbitrary units, a.u.) from individual CXCR4-AcGFP trajectories in cells treated as indicated. The mean of all trajectories is indicated (n.s. not significant; *p ≤ 0.05; ****p ≤ 0.0001). **e** Frequency of CXCR4-AcGFP receptors present in particles containing more than 10 receptors expressed as a percentage of the total receptors present in particles containing ≥ 3 receptors and calculated as in (D)

Recent studies have shown a link between chemokine-mediated directed cell migration and the ability of the ligand to promote receptor nanoclustering at the cell membrane [25, 34]. Using TIRF microscopy and SPT of CXCR4-AcGFP in transiently transfected JKX4^−/−^ cells [34], we examined the effect of ChOx treatment on CXCR4 dynamics, both at steady-state and following CXCL12 stimulation (Supplemental video 3–6). We first established appropriate expression conditions for detecting and tracking individual CXCR4 spots, as described [25]. Trajectory analysis [43] revealed that ChOx treatment modulated CXCR4 dynamics under steady-state conditions and after CXCL12 activation. At steady-state, a high proportion of CXCR4 particles were mobile in both experimental conditions (~ 92% in untreated cells vs. ~ 93% in ChOx-treated cells). CXCL12 activation increased the immobile fraction in both cases, but the effect was less pronounced in ChOx-treated cells (~ 29% in control cells vs. ~ 19% in ChOx-treated cells) (Fig. 6a). While the median short time-lag diffusion coefficient (D_1−4_) for CXCR4 trajectories was similar in steady-state conditions in both cases (0.017 µm^2^s^–1^ in untreated cells vs. 0.019 µm^2^s^–1^ in ChOx-treated cells), ChOx-treated cells showed higher D_1−4_ after CXCL12 activation than activated controls (untreated, median D_1−4_ = 0.0073 µm^2^s^–1^ vs. ChOx-treated, median D_1−4_ = 0.0089 µm^2^s^–1^) (Fig. 6b). To estimate receptor numbers per trajectory, we measured the average fluorescence intensity of the first 20 frames of each trajectory, using the intensity of monomeric CD86-AcGFP as a reference [50]. At steady-state, CXCR4 existed predominantly as monomers and dimers in both untreated and ChOx-treated cells, with a higher proportion of monomers and dimers in ChOx-treated cells (~ 79% for control vs. ~ 90% for ChOx-treated cells) (Fig. 6c), consistent with lower basal MSI for CXCR4 in both cases (98.04 a.u. in control vs. 78.80 a.u. in ChOx-treated cells) (Fig. 6d). As previously reported [25], CXCL12 activation promoted CXCR4 membrane nanoclustering in both groups (~ 55% and 53% of receptors present in particles containing ≥ 3 receptors) (Fig. 6c). Focusing on larger clusters (≥ 10 receptors/particle), we observed that ChOx treatment attenuated CXCL12-induced clustering (~ 23% for control vs. ~ 11% for ChOx-treated cells) (Fig. 6e). These findings indicate that a moderate reduction in cholesterol levels permits CXCL12-mediated CXCR4 clustering but alters the spatiotemporal regulation of CXCR4 nanoclustering and dynamics.

#### ChOx treatment does not disrupt membrane microdomains

**Fig. 7:**
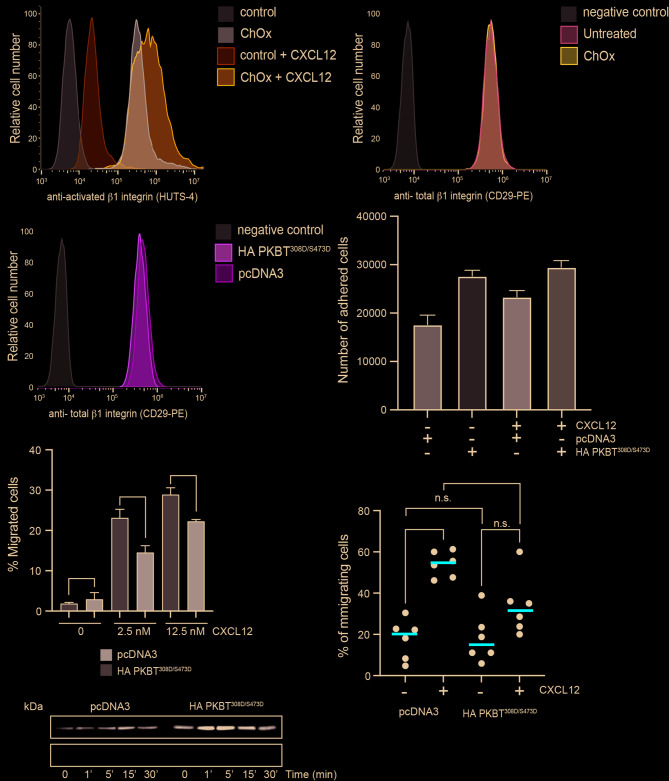
Effect of cholesterol oxidase and bacterial sphingomyelinase on GM1 lipid rafts.** a** Confocal analysis of Jurkat cells untreated or treated with ChOx or bSMase and plated on fibronectin. Cells were stained with CTxB-FITC (a lipid raft marker GM1) and with AF546-anti-CXCR4 mAb (clone 44717). Figure also includes the visible image (brightfield) and overlay panels of the three channels (*n* = 3). Scale bar, 10 μm. **b** Quantification of the percentage of cells containing a patchy profile of CTxB staining versus the total cells evaluated in each experimental conditions (*n* = 3, n.s. not significant; ***p ≤ 0.001)

Membrane microdomains are specific subdomains within membranes enriched in cholesterol and/or sphingolipids that serve as platforms for protein-lipid and protein-protein interactions. Studies have suggested that they play essential roles in chemokine function. Indeed, the association between CXCR4 and lipid-enriched membrane microdomains is functionally important [28, 29]. We previously reported that prolonged treatment of T cells with bSMase, which breaks down sphingomyelins and leads to ceramide accumulation, disrupts CXCR4 clustering and alters CXCL12-mediated directed migration of T cells [10]. Therefore, we investigated whether the reduction in cholesterol levels caused by ChOx treatment similarly affects membrane microdomains. Jurkat cells were untreated, treated with ChOx, or treated with bSMase (as a control), fixed, and then stained with CTxB-FITC (to visualize GM1 lipid rafts) and an AF546-anti-CXCR4 mAb. In the majority of untreated cells, CTxB staining showed a patchy distribution in the cell membrane, with strong co-localization between CTxB and anti-CXCR4 staining (Fig. 7a, b). Notably, ChOx treatment did not alter this patchy distribution or the co-localization of CTxB and CXCR4 (Fig. 7a, b). As expected, bSMase treatment, which disrupts sphingomyelins, completely abolished the patchy CTxB distribution at the cell membrane (Fig. 7a, b). These results suggest that while ChOx treatment may slightly reduce cholesterol levels, it does not significantly disrupt membrane microdomains.

## Discussion

CXCR4, a well-studied chemokine receptor, is activated by its ligand CXCL12, and primarily signals through Gi protein coupling [51]. This signaling cascade regulates cell migration in diverse physiological processes such as hematopoiesis, neovascularization, and angiogenesis [52]. CXCR4 is also implicated in numerous diseases [53], including tumorigenesis and cancer metastasis [54]. Additionally, it functions as a co-receptor for X4-tropic human immunodeficiency virus (HIV-1) strains [55]. CXCR4 exists in dynamic equilibrium between monomeric, dimeric, and higher-order oligomeric states, all of which are crucial for CXCL12-mediated cell responses [25, 56]. As an integral membrane protein, CXCR4 interacts with both membrane lipids and proteins, which ultimately dictate its dynamic structure and function. Molecular dynamics simulations suggest that cholesterol binding disrupts the dimer formation favored by CXCR4 in pure phospholipid bilayers [20]. Using two different energy transfer-based techniques, FRET and FLIM, we found that ChOx treatment induces conformational changes in CXCR4. Notably, despite these structural changes, CXCR4 retained its capacity to bind CXCL12. This observation is supported by the identification of a cholesterol molecule situated between CXCR4 protomers, engaging a series of hydrophobic residues [32]. Moreover, sphingomyelin depletion in T cells has been shown to downmodulate CXCL12-mediated CXCR4 oligomerization at the cell membrane, thereby abolishing ligand-mediated directed migration [10]. Lipidomic analysis revealed a modest but significant reduction in some sphingomyelin species. This reduction might contribute to the impaired cell migration observed in ChOx-treated cells; however, further experiments are needed to test this hypothesis, especially given the lack of correlation between sphingomyelin downregulation and upregulation of corresponding ceramide species.

To investigate the impact of cholesterol depletion on CXCR4 lateral mobility and functions, we initially employed MCD, a commonly used cholesterol-depleting agent, as described in several studies [30, 57–60]. However, MCD treatment not only perturbed CXCR4-mediated functions but also altered receptor conformation, preventing the binding of two conformational mAbs and the chemokine CXCL12. This disruption of ligand binding has also been observed with other GPCRs [61–63]. Recognizing that MCD treatment can have variable effects depending on cell type, concentration, and exposure time [64], we explored an alternative approach. ChOx, a microbial flavoenzyme belonging to the oxidoreductase family [65], catalyzes the oxidation of cholesterol to cholestenone. Unlike cholesterol, cholestenone does not effectively promote membrane ordering [66]. ChOx treatment offers the advantage of modifying cholesterol without extracting it from the cells, thus minimizing cellular stress. Lipidomic analyses confirmed that ChOx induced a much smaller reduction in cholesterol compared with MCD. Critically, ChOx treatment did not impair CXCL12 binding or signaling pathways. In ChOx-treated cells, CXCL12 triggered Ca^2+^ flux normally and activated ERK1/2 and PI3K pathways. However, we observed sustained and increased Akt phosphorylation at residue Thr308 compared with controls. Whereas p-Ser473 modulates Akt activity, p-Thr308 is required for its catalytic activity [67]. Although further investigation is required, this result suggests that ChOx treatment might affect negative regulators of Akt activity. Although no changes were observed in total β1-integrin content, cell adhesion increased in Jurkat cells transiently transfected with a constitutively active AKT mutant, which exhibits strong and sustained AKT phosphorylation. Despite normal signaling, ChOx treatment abolished the ability of cells to sense CXCL12 gradients in a direct-migration chamber. Cell migration is a complex process requiring adhesion, migration, and actin cytoskeleton reorganization [68]. Chemotaxis requires cell polarization, with lamellipodia formation at the leading edge (where chemokine receptors concentrate) and uropod retraction at the trailing edge where focal adhesions are disassembled to allow the cell body to move forward [69, 70]. Interestingly, ChOx treatment did not affect CXCL12-induced actin polymerization or cell polarization.

Analysis of the effects of ChOx on CXCL12-mediated directional cell migration indicated reduced motility, suggesting a potential increase in cell adhesion to fibronectin. This was supported by flow cytometry data showing a higher proportion of active β1-integrins in ChOx-treated cells, detected using a mAb specific to the active β1-integrin conformation, without changes to the total amount of β1-integrins. In addition, Jurkat cells transiently transfected with a constitutive active AKT mutant, HA PKB^T308D/S473D^, showed, after CXCL12 stimulus, increased cell adhesion to fibronectin and reduced migration on integrin substrates. These data suggest a potential link between AKT hyperphosphorylation, β1 integrin activation and the inability of cells to migrate towards chemokine gradients. Previous studies have shown that cholesterol depletion affects cell adhesion to integrin substrates [71, 72]. While further experiments are underway to investigate these findings, the existing literature indicates that cholesterol influences the lateral mobility of integrins [73], and that an oxysterol metabolite of cholesterol can directly interact with integrins to promote focal adhesion activation [74].

We have recently described that CXCL12-mediated receptor oligomerization is crucial for proper cell orientation in chemokine gradients. CXCR4^R334X^, a truncated mutant chemokine receptor associated with WHIM syndrome (warts, hypogammaglobulinemia, infections, myelokathexis), exhibits impaired CXCL12-induced nanoclustering, altered lateral mobility and spatial organization, and failed to promote directed-cell migration [34]. In the present study, moderate cholesterol depletion using ChOx perturbed CXCL12-induced CXCR4 clustering dynamics. While CXCL12-mediated CXCR4 clustering was still observed following ChOx treatment, the proportion of receptors incorporated into larger clusters (containing ≥ 10 receptors/particle) was reduced. This correlated with a decrease in the immobile fraction of CXCR4 particles and an increase in its diffusion coefficient. Although the precise relationship between the number of receptors per cluster and directed cell migration remains unclear, it is known that CCR7 oligomerization influences cell migration efficiency, and a super-oligomerizing mutant (CCR7^V317I^) exhibiting enhanced migration has been described [60].

CXCR4, like other chemokine receptors, resides in rigid and liquid-ordered membrane microdomains enriched in sphingomyelins, glycosphingolipids, and cholesterol [75, 76]. Our lipidomic analysis demonstrated that ChOx had a limited effect on cholesterol levels in Jurkat cells. Cholesterol within cell membranes is generally a poor substrate for ChOx, likely due to inefficient access to these microdomains [77]. Furthermore, sphingomyelins present in membrane microdomains can hinder ChOx access to cholesterol [78, 79]. We thus used CTxB and anti-CXCR4 immunocytochemistry analysis to determine the effect of ChOx or bSMase (control) on microdomain structure. In both untreated and ChOx-treated cells, CTxB (which binds to GM1 ganglioside and cholesterol-rich domains) exhibited a patchy distribution, indicative of intact microdomains [80], and CXCR4 co-localized with CTxB. However, treatment with bSMase completely disrupted this distribution, resulting in uniform CTxB staining. These data suggest that whereas sphingomyelin depletion disrupts the structure of lipid microdomains, ChOx treatment has a comparatively milder effect. Consistent with our observations, in vitro studies using two-dimensional and three-dimensional lipid models have shown that ChOx treatment increases membrane fluidity and flexibility, while maintaining membrane stability [81]. We hypothesize that ChOx has limited access to cholesterol within lipid rafts, leading to a modulation of membrane fluidity without gross disruption of the microdomains themselves. Consequently, while CXCL12-mediated CXCR4 oligomerization is still observed, the clusters are smaller. Conversely, bSMase treatment, by depleting sphingomyelins, disrupts lipid microdomains and abrogates ligand-mediated CXCR4 nanoclustering [10].

Despite observing no defects in ligand binding, receptor activation, or cell polarization, cells treated with ChOx were unable to sense CXCL12 in directional cell migration assays. Previous work has shown that cholesterol depletion can significantly increase membrane-cytoskeleton adhesion energy [82] and affect various cellular processes, including exocytosis, endocytosis, lamellipodial retraction and extension, and cell migration [83]. Although the effects are less pronounced at 37 °C, cholesterol depletion reduces the viscosity of the membrane surface and disassembles the actin network, affecting the mechanical properties of a cell through the cytoskeleton [82].

Altogether, our data indicate that moderate cholesterol depletion, while preserving lipid microdomains, alters CXCR4 oligomer distribution, reducing the proportion of receptors present in large clusters and increasing active β1-integrins. These changes may contribute to the observed defects in CXCL12 gradient sensing. Given the challenges in directly targeting chemokine receptors in health and disease, our findings highlight the potential for modulating chemokine function by targeting the membrane environment in which these receptors reside.

## Conclusions

We provide evidence that membrane cholesterol influences the lateral mobility and spatial organization of the chemokine receptor CXCR4 on the cell surface, as well as the ability of cells to sense chemoattractant gradients. Critically, ChOx treatment affected CXCR4 conformation and membrane fluidity, but did not significantly affect ligand binding or other chemokine-mediated signaling pathways. Our findings highlight the physiological importance of maintaining membrane integrity for proper chemokine receptor function and demonstrate the possibility of modulating chemokine function by altering the membrane environment, rather than directly targeting the receptor-binding site.

## Supplementary Information

Supplementary Material 1

Supplementary Material 2

Supplementary Material 3

Supplementary Material 4

Supplementary Material 5

Supplementary Material 6

Supplementary Material 7

Supplementary Material 8

Supplementary Material 9

## Data Availability

No datasets were generated or analysed during the current study.

## Abbreviations

bSMase: Bacterial sphingomyelinase
ChOx: cholesterol oxidase
CTxB: Cholera Toxin B
GPCR: G protein-coupled receptor
D_1−4_: Diffussion Coefficient
FMI: Forward Migration Index
FRET: Fluorescence resonance energy transfer
GFP: Green Fluorescent Protein
JKX4^−/−^: Jurkat cells lacking endogenous CXCR4
JKX4^−/−^-X4: JKX4^−/−^ transfected with CXCR4-GFP
MCD: methyl-β-cyclodextrin
MSI: Mean Spot Intensity
RICS: Raster Image Correlation Spectroscopy
SPT: Single-particle tracking
TIRF: total internal reflection fluorescence
TM: Transmembrane
