# Supplementary material for: Cholesterol oxidase treatment impairs CXCR4-mediated T cell migration
Source: Cell Commun Signal. 2025 Oct 17;23:444. doi: 10.1186/s12964-025-02392-9 (PMC12532918; doi:10.1186/s12964-025-02392-9)
Supplement: Supplementary file 1 — Supplementary Material 1 [file 12964_2025_2392_MOESM1_ESM.pdf]

pErk(1/2)

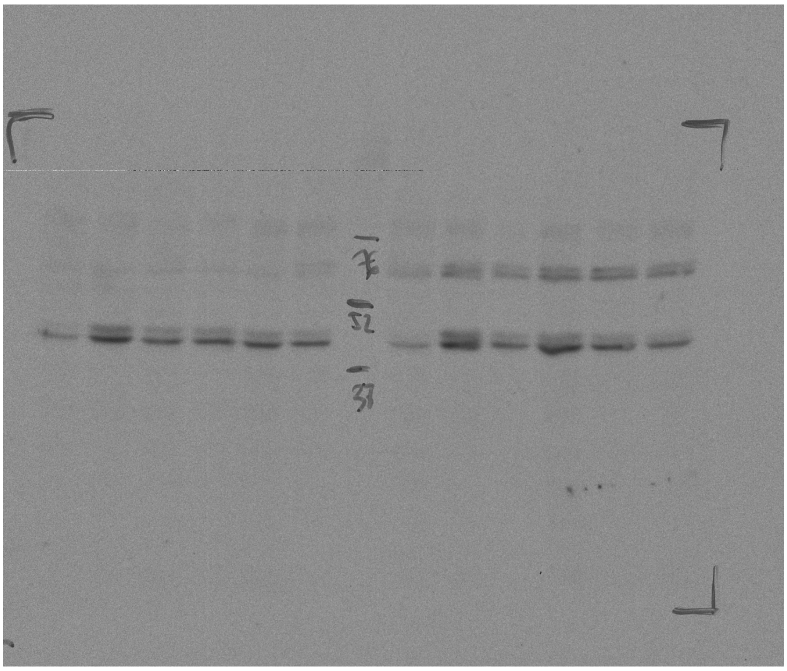

Erk(1/2)

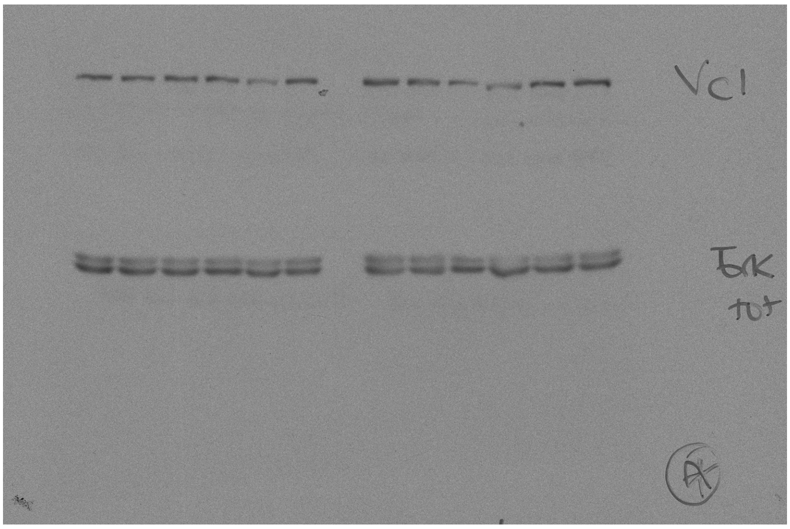

Full uncropped Blots image(s) related to Figure 2D. Figure shows the band corresponding to pErk(1/2), upper panel, and total Erk(1/2), lower panel. Additional bands correspond to other antibodies employed previously in the same western.

pAkt (T308)

Akt

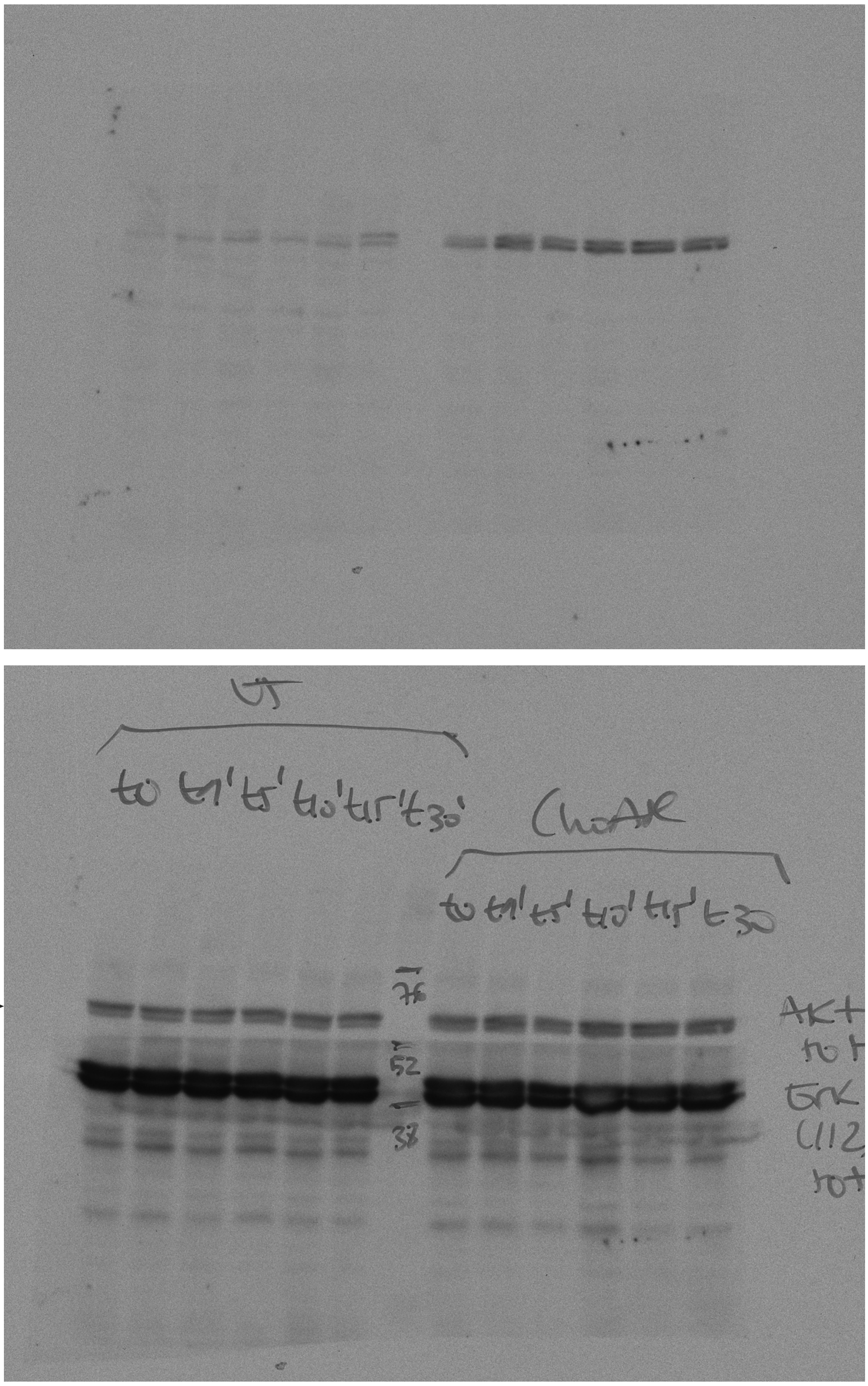

Full uncropped Blots image(s) related to Figure 2D. Figure shows the band corresponding to pAkt (T308), upper panel and total Akt, lower panel. Additional bands correspond to other antibodies employed previously in the same western.

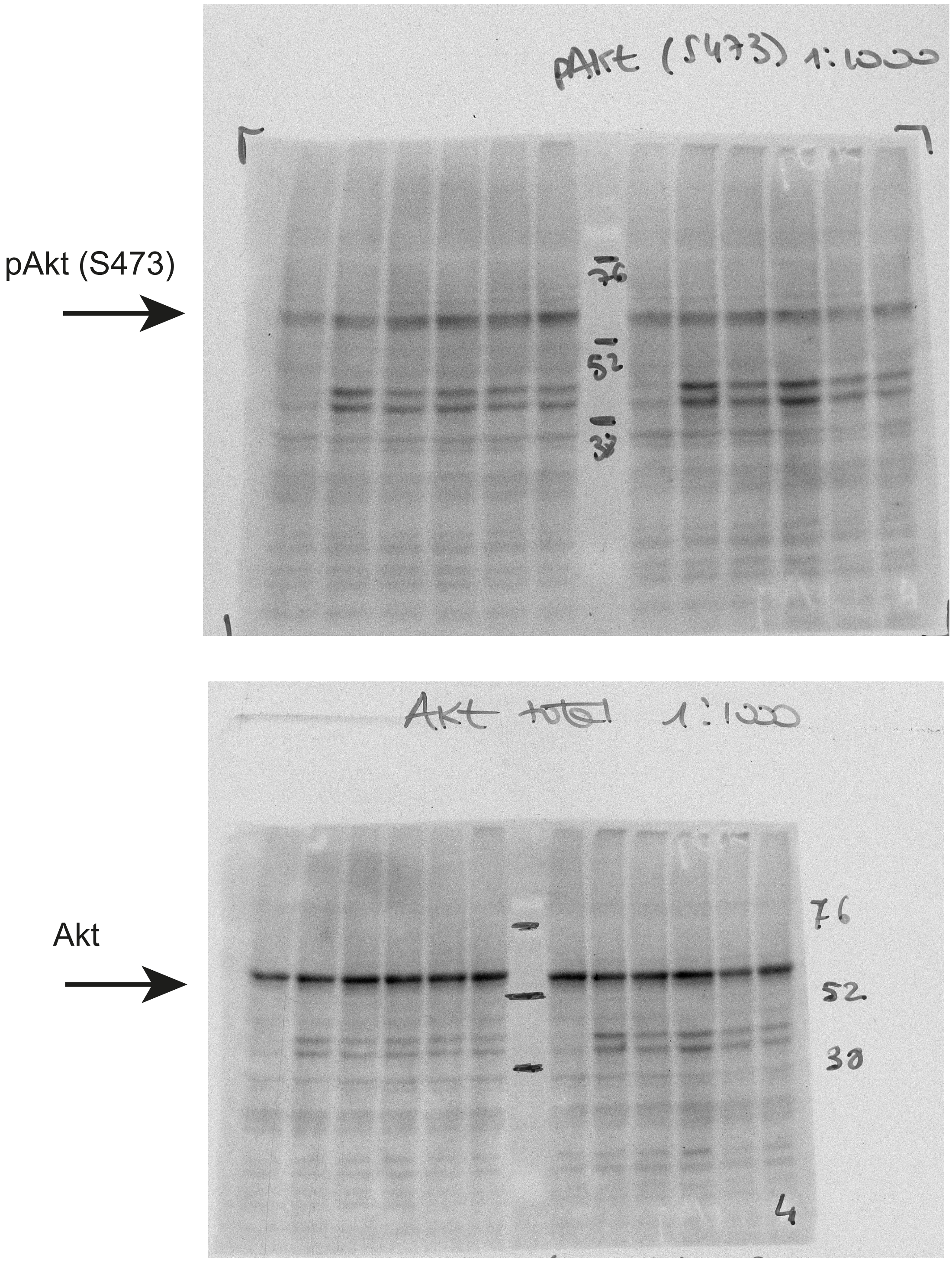

Full uncropped Blots image(s) related to Figure 2D. Figure shows the band corresponding to pAkt (S473), upper panel, and total Akt, lower panel. Additional bands correspond to other antibodies employed previously in the same western.
