# Supplementary material for: Cholesterol oxidase treatment impairs CXCR4-mediated T cell migration
Source: Cell Commun Signal. 2025 Oct 17;23:444. doi: 10.1186/s12964-025-02392-9 (PMC12532918; doi:10.1186/s12964-025-02392-9)
Supplement: Supplementary file 2 — Supplementary Material 2 [file 12964_2025_2392_MOESM2_ESM.pdf]

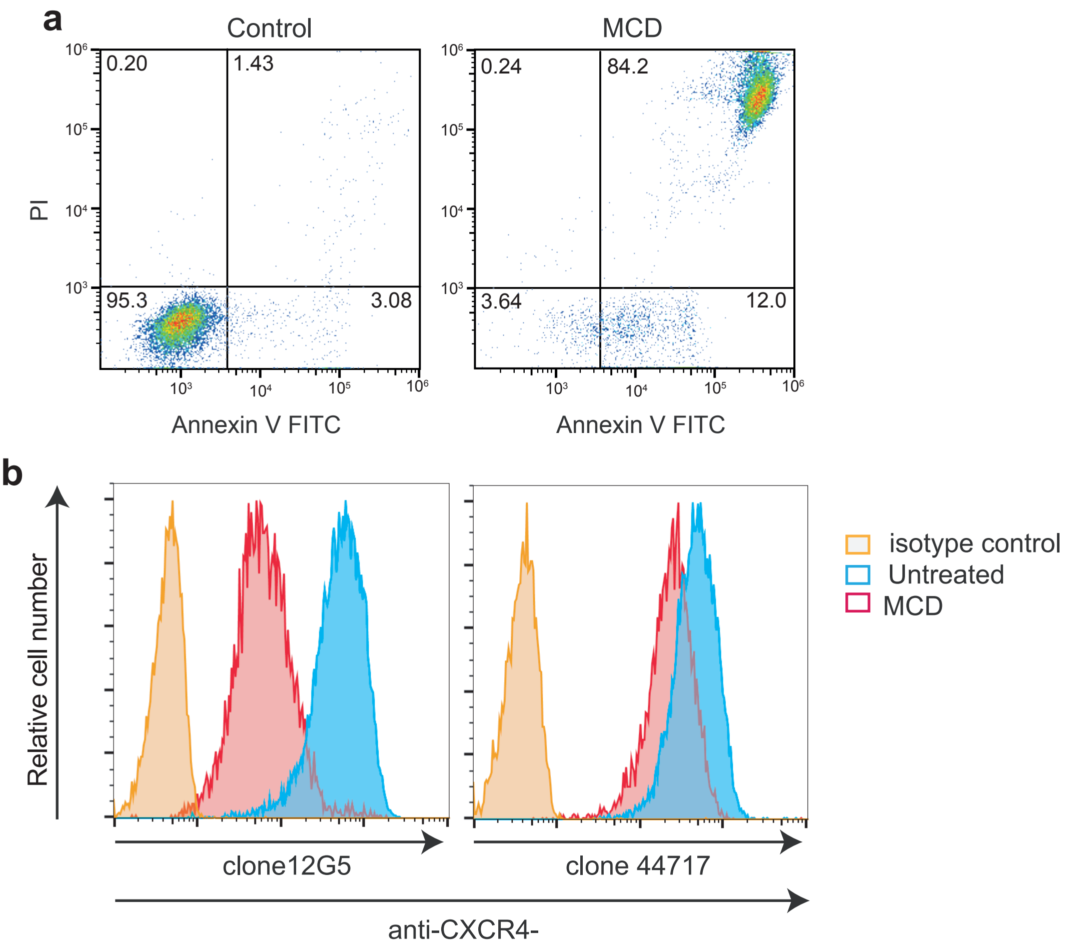

Supplementary Figure 1. Cell viability and CXCR4 expression in Jurkat cell untreated and treated with MCD. (**a**) Flow cytometry biparametric histograms of shape versus complexity (FSC/SSC) of untreated and MCD-treated Jurkat cells. Panels include the percentage of viable cells in each condition. A representative experiment of 4 is shown. (**b**) CXCR4 expression at the surface of untreated and MCD-treated Jurkat cells analyzed by flow cytometry using two distinct anti-CXCR4 conformational antibodies (clones 12G5 and 44717). Data are from a representative experiment of 3 performed.

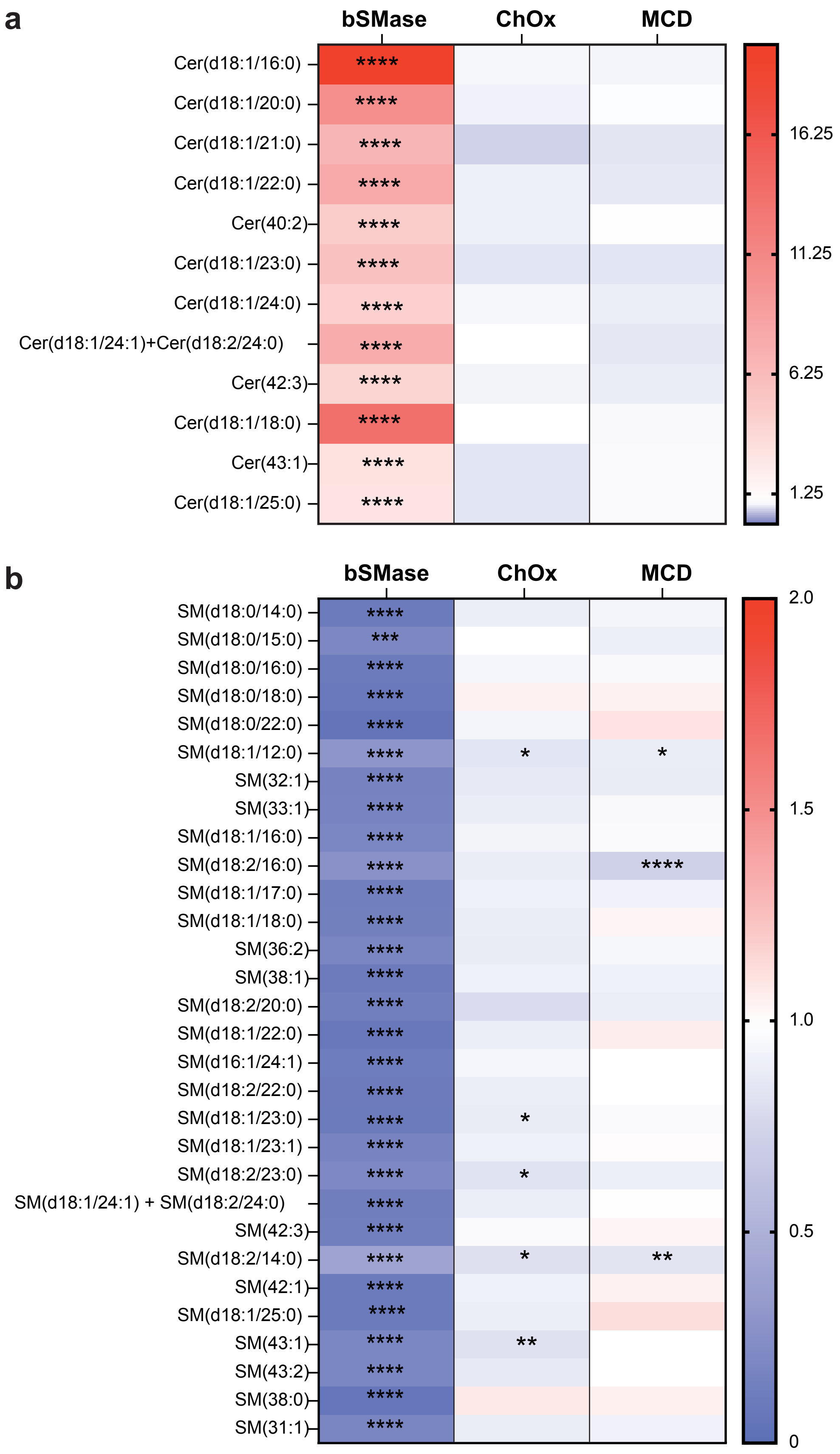

Supplementary Figure 2. Lipidomic analysis using UHPLC-MS of Jurkat cells untreated or treated with bSMase, ChOx, or MCD. Heatmap representing binary comparisons of Jurkat cells treated with bSMase, ChOx, or MCD compared with the control (untreated) group per metabolite. Results show the relative increase (red) or decrease (blue) in distinct ceramides (**a**) or sphingomyelin species (**b**). Heatmap color codes for log2 (fold-change) are shown. Student's t-test (n = 4, *p ≤ 0.05; **p ≤ 0.01; ****p ≤ 0.0001).

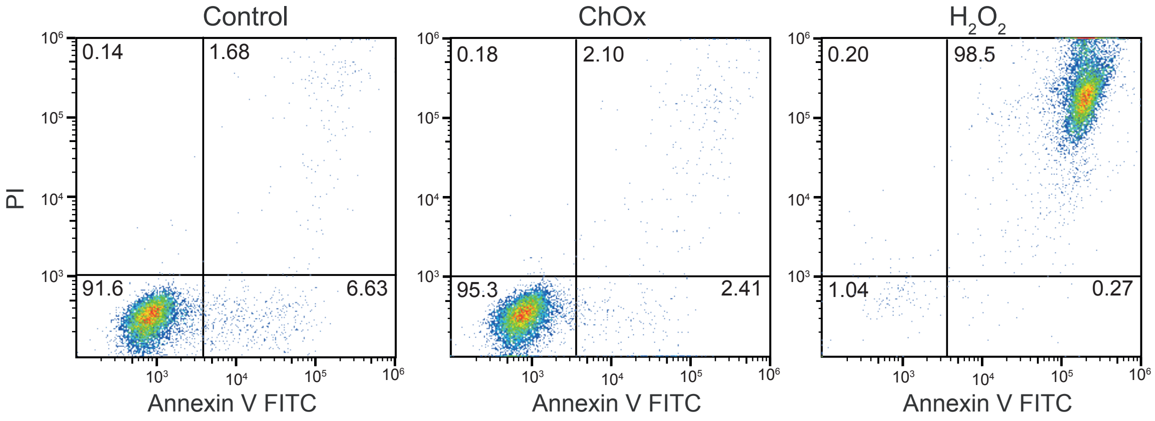

Supplementary Figure 3. Jurkat cell viability. Viability of Jurkat cells untreated and treated with ChOx (25 U/ml, 2 h, 37 ºC) as determined by Annexin-V/PI incorporation and flow cytometry analysis. As a positive control for cell death, Jurkat cells were treated with 10% H2O2. Panels include the percentage of cells in each condition. A representative experiment of 3 independent experiments is shown.

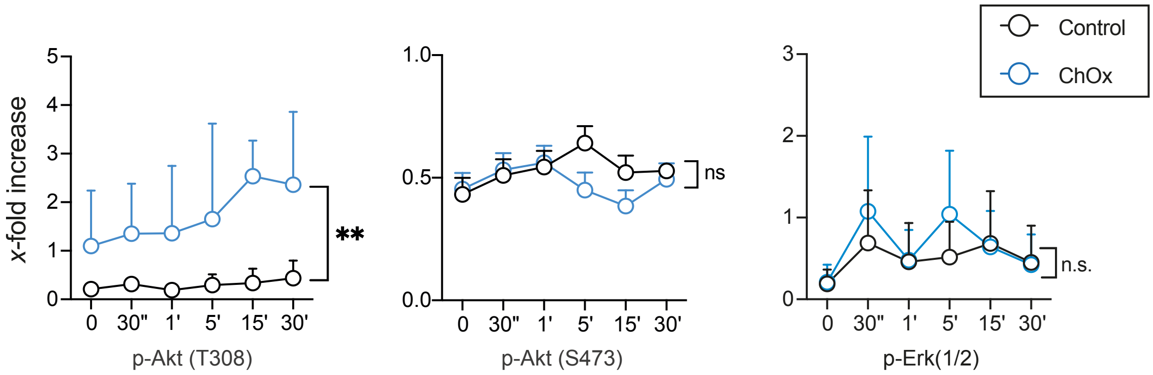

Supplementary Figure 4. Densitometry analysis of the western blot images in Figure 5D using Image J. Western blot analysis of ERK 1/2 and Akt phosphorylation at residues T308 and S473 in untreated and ChOx-treated Jurkat cells stimulated with CXCL12 (50 nM) at the indicated time points. Membranes were reblotted with anti-Akt and anti-ERK antibodies as a loading control.(n=3; n.s., not significant; ** $p \leq 0.01$).
