# Supplementary material for: Cholesterol oxidase treatment impairs CXCR4-mediated T cell migration
Source: Cell Commun Signal. 2025 Oct 17;23:444. doi: 10.1186/s12964-025-02392-9 (PMC12532918; doi:10.1186/s12964-025-02392-9)
Supplement: Supplementary file 3 — Supplementary Material 3 [file 12964_2025_2392_MOESM3_ESM.pdf]

Supplementary Video 1 (separate file). Related to Figure 3A. Representative video of untreated Jurkat cell migration on fibronectin-coated μ-Slide Chemotaxis chambers following a CXCL12 gradient on top. Images over time (15 frames/s) are shown. Overlaid trajectories of cells shown in movie were detected and tracked using Fiji software.

Supplementary Video 2 (separate file). Related to Figure 3A. Representative video of ChOx-treated Jurkat cell migration on fibronectin-coated μ-Slide Chemotaxis chambers following a CXCL12 gradient on top. Images over time (15 frames/s) are shown. Overlaid trajectories of cells shown in movie were detected and tracked using Fiji software.

Supplementary Video 3 (separate file). Related to Figure 6. Representative video of CXCR4-AcGFP on live JKCD4^{+}X4^{-} cells captured by SPT-TIRF, showing the diffusion of CXCR4 particles (monomers, dimers, and nanoclusters) at steady state (FN). The video was acquired and displayed at 10 frames/s.

Supplementary Video 4 (separate file). Related to Figure 6. Representative video of CXCR4-AcGFP on live JKCD4^{+}X4^{-} cells captured by SPT-TIRF, showing the diffusion of CXCR4 particles (monomers, dimers, and nanoclusters) in response to CXCL12. The video was acquired and displayed at 10 frames/s.

Supplementary Video 5 (separate file). Related to Figure 6. Representative video of CXCR4-AcGFP on live JKCD4^{+}X4^{-} cells treated with ChOx and captured by SPT-TIRF, showing the diffusion of CXCR4 particles (monomers, dimers, and nanoclusters) at steady state (FN). The video was acquired and displayed at 10 frames/s.

Supplementary Video 6 (separate file). Related to Figure 6. Representative video of CXCR4-AcGFP on live JKCD4^{+}X4^{-} cells treated with ChOx and captured by SPT-TIRF, showing the diffusion of CXCR4 particles (monomers, dimers, and nanoclusters) in response to CXCL12. The video was acquired and displayed at 10 frames/s.
